# PRMT5: A novel regulator of Hepatitis B virus replication and an arginine methylase of HBV core

**DOI:** 10.1371/journal.pone.0186982

**Published:** 2017-10-24

**Authors:** Barbora Lubyova, Jan Hodek, Ales Zabransky, Hana Prouzova, Martin Hubalek, Ivan Hirsch, Jan Weber

**Affiliations:** 1 Institute of Organic Chemistry and Biochemistry of the Czech Academy of Sciences, IOCB & Gilead Research Center, Prague, Czech Republic; 2 Dept. Genetics and Microbiology, Faculty of Sciences, Charles University, Prague, Czech Republic; Indiana University, UNITED STATES

## Abstract

In mammals, protein arginine methyltransferase 5, PRMT5, is the main type II enzyme responsible for the majority of symmetric dimethylarginine formation in polypeptides. Recent study reported that PRMT5 restricts Hepatitis B virus (HBV) replication through epigenetic repression of HBV DNA transcription and interference with encapsidation of pregenomic RNA. Here we demonstrate that PRMT5 interacts with the HBV core (HBc) protein and dimethylates arginine residues within the arginine-rich domain (ARD) of the carboxyl-terminus. ARD consists of four arginine rich subdomains, ARDI, ARDII, ARDIII and ARDIV. Mutation analysis of ARDs revealed that arginine methylation of HBc required the *wild-type* status of both ARDI and ARDII. Mass spectrometry analysis of HBc identified multiple potential ubiquitination, methylation and phosphorylation sites, out of which lysine K7 and arginines R150 (within ARDI) and R156 (outside ARDs) were shown to be modified by ubiquitination and methylation, respectively. The HBc symmetric dimethylation appeared to be linked to serine phosphorylation and nuclear import of HBc protein. Conversely, the monomethylated HBc retained in the cytoplasm. Thus, overexpression of PRMT5 led to increased nuclear accumulation of HBc, and *vice versa*, down-regulation of PRMT5 resulted in reduced levels of HBc in nuclei of transfected cells. In summary, we identified PRMT5 as a potent controller of HBc cell trafficking and function and described two novel types of HBc post-translational modifications (PTMs), arginine methylation and ubiquitination.

## Introduction

Hepatitis B virus (HBV) is a human pathogen that chronically infects more that 240 million people worldwide [1, 2]. Chronically infected individuals are at high risk of developing chronic liver diseases, including chronic hepatitis, cirrhosis, and hepatocellular carcinoma (HCC) [3, 4]. The genome of HBV consists of circular partially double-stranded DNA, which is approximately 3.2 kb long and encodes four genes designated C (core), X, P (polymerase) and S (surface).

The full-length HBV core protein (HBc) is a 21.5 kDa protein consisting of 183 (ayw subtype) or 185 (adw subtype) amino acids. It contains two distinct domains, the N-terminal self-assembly domain (amino acids 1–140) and the arginine-rich C-terminal domain (ARD, amino acids 150–185), which possesses nucleic acid-binding properties [5, 6]. The HBc protein is a phosphoprotein with three major phosphorylated serine residues 157, 164, 172 [6–8]. Phosphorylation of HBc correlates with its nucleus-to-cytoplasm shuttling, pre-genomic RNA (pgRNA) encapsidation, reverse transcription, and viral transport [9–12].

The ARD of HBc contains four stretches of clustered arginines, ARDI, ARDII, ARDIII and ARDIV. Li et al. [13] recently reported that ARDI and ARDIII are associated with two co-dependent nuclear localization signals (NLS), while ARDII and ARDIV behave like two independent nuclear export/cytoplasm retention signals (NES/CRS). The nuclear export of HBc protein was shown to be dependent on the NFX1-p15 pathway [14]. Clinically, cases of chronic hepatitis manifesting severe liver damage and inflammation are associated with cytoplasm-predominant HBc [15, 16].

There is a growing evidence that arginine methylation is an important type of post-translational modification that plays a role in a variety of biological processes including chromatin regulation, transcription control, RNA processing and nuclear transport [17–21]. Arginine methylation has been shown to be enriched on RNA-binding proteins [22, 23]. Indeed, over 50% of arginine methylation found in mammalian cells is concentrated on heterogeneous nuclear ribonucleoproteins (hnRNP) [24]. In addition, a number of well-characterized methylation sites are found on histone tails and splicing factors [25, 26]. Arginine methylation is catalyzed by two major groups of protein arginine methyltransferases (PRMTs). Type I enzymes (PRMT1,2,3,4,6, and 8) catalyze production of monomethyl arginine (MMA) and asymmetric dimethylarginine (aDMA) and type II PRMTs (PRMT5,7,9) generate MMA and symmetric dimethylarginine (sDMA) [27–29]. PRMT5 is the primary and well described type II arginine methyltransferase. PRMT5 is usually found in a complex with the WD-repeat protein, methylosome protein 50—MEP50, (also known as WDR77) that greatly enhances its methyltransferase activity through increased affinity for the protein substrate [30]. PRMT5’s substrate proteins include myelin basic protein, histones, and the spliceosomal Sm proteins [19, 31–33].

It is noteworthy that in previous studies, the type I enzyme, PRMT1, was implicated as a negative regulator of HBV transcription [34]. HBV X protein (HBx) was shown to directly associate with PRMT1 and inhibit PRMT1-mediated protein methylation. Very recently, Zhang et al. [35] published similar observations regarding the PRMT5-mediated inhibition of HBV replication. They showed that PRMT5 represses HBV through epigenetic regulation of cccDNA transcription and interference with pre-genomic RNA encapsidation.

To explore more general role of PRMTs in HBV replication, we hypothesized that a common cellular regulatory pathway may control HBV growth and replication. We tested whether other PRMTs, their splicing variants and co-factors also influence HBV transcription and replication. Here, we identified PRMT5 splicing variants v1 and v2 (PRMT5v1 and PRMT5v2) as effective regulators of HBV replication affecting the total level of HBV DNA, cccDNA pools as well as pre-genomic RNA expression. While overexpression of PRMT3 and PRMT5’s co-factor MEP50 led to reduction of HBV replication, their depletion did not show an inverse effect. Furthermore, we found that PRMT5 specifically interacts with HBc protein, allowing symmetric dimethylation of HBc. Symmetrically dimethylated HBc predominantly localizes to nuclei of transfected cells. In contrast, arginine monomethylation of HBc protein occurs exclusively on cytoplasmic HBc. Thus, our data and the recent reports show that in addition to PRMT1, also PRMT5 exhibits the regulatory role in HBV replication and define arginine methylation as another type of post-translational modification, which may affect the function of HBc protein. Concomitant effect of PRMT5 on HBc function and cccDNA epigenetic regulation can potentiate both mechanisms and make of PRMT5 an important restriction factor of HBV replication.

## Results

### PRMT5 negatively regulates HBV replication

**Fig 1:**
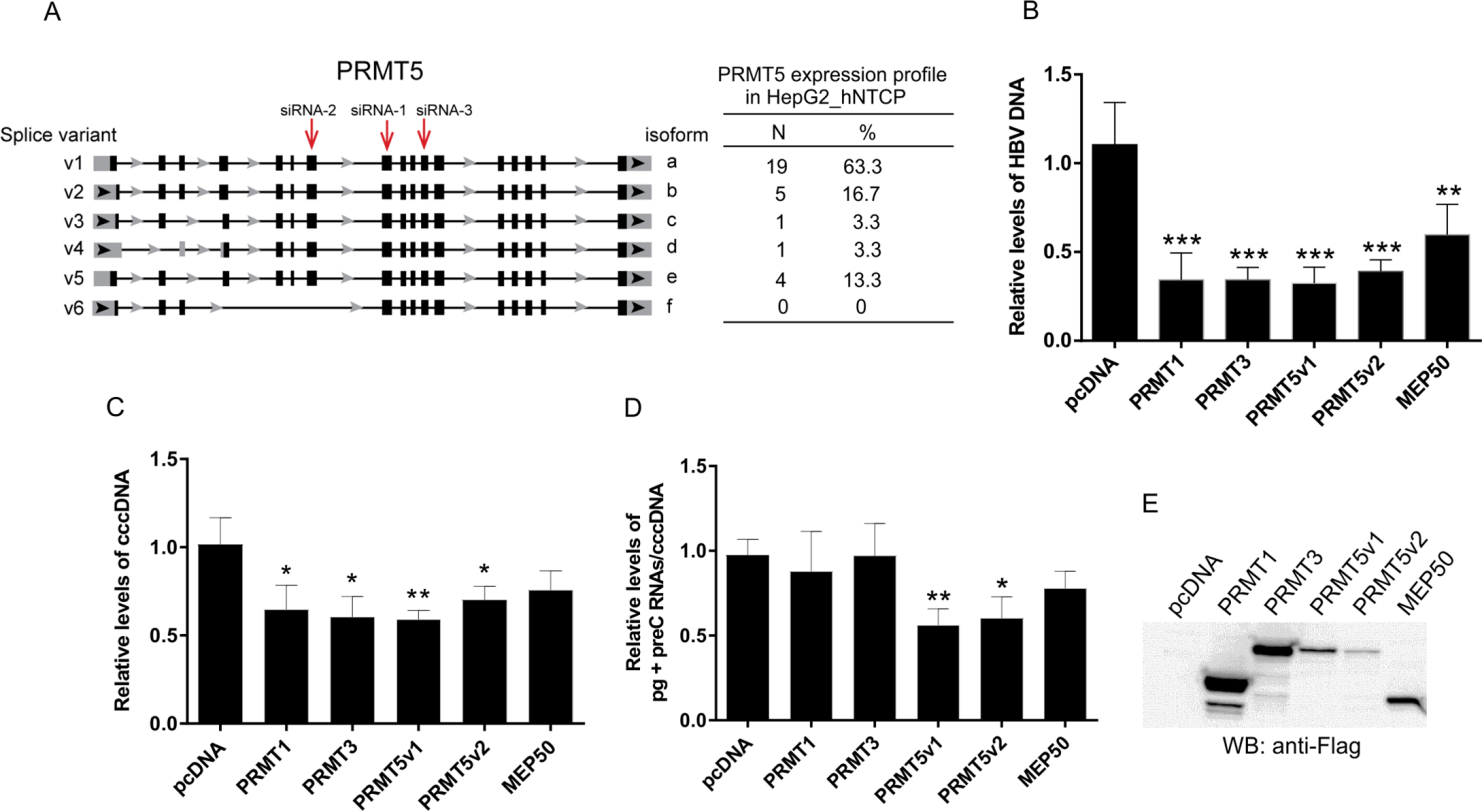
PRMT5 represses HBV replication and transcription. (A) Schematic representation of PRMT5 splicing variants (based on NCBI human genome sequence of PRMT5 from www.ncbi.nlm.nih.gov), their expression profile in HepG2_hNTCP cells and positions of PRMT5-specific siRNAs used in this work. N, number of clones corresponding to PRMT5 splice variants. (B) and (C) HepG2_hNTCP cells were transfected with expression plasmids for PRMT1, PRMT3, PRMT5v1, PRMT5v2 and MEP50/WDR77 tagged with Flag, or an empty vector, pcDNA. Forty-eight hours after transfection, cells were infected with HBV (1000 VGE/cell). Four days post-infection, cells were harvested for DNA and RNA isolation. The quantification of total HBV DNA (B) and cccDNA (C) were analyzed by qPCR and the levels were normalized to albumin. (D) HBV RNA (pgRNA+preC RNA) transcription was analyzed by RT-qPCR. The RNA transcript levels were normalized to cccDNA. Asterisks indicate statistically significant differences between the control (pcDNA) and PRMTs groups determined by ANOVA (* P<0.05; ** P<0.01; *** P<0.001). Error bars represent standard deviations (SD) of three independent experiments. (E) The expression levels of transfected PRMT1, 3, 5 and MEP50 were estimated by Western blotting using anti-Flag antibodies.

Recently, Benhenda et al. [34] identified PRMT1 as a binding factor of HBx and an inhibitor of HBV transcription. More recently, Zhang et al. [35] reported that PRMT5 restricts HBV replication by epigenetic mechanism. We tested whether other PRMTs, their splicing variants and co-factors also influence HBV transcription and replication. The PRMTs which were included in our assays were both type I (PRMT1 and PRMT3) and type II (PRMT5 along with its co-factor MEP50/WDR77). Since PRMT5 gene is known to produce multiple splicing variants (v1 to v6, Fig 1A) encoding isoforms, a to f, we first investigated the expression profile of PRMT5 isoforms in the hepatocyte cell line, HepG2_hNTCP. Semi-quantitative low-cycle-number PCR after reverse transcription (RT-PCR) showed that the HepG2_hNTCP cells expressed three major PRMT5 splicing variants v1 (isoform a), v2 (isoform b) and v5 (isoform e), which comprise about 63.3% (19/30), 16.7% (5/30) and 13.3% (4/30) of all transcripts detected, respectively (Fig 1A). Two isoforms, v3 and v4, collectively represented 6.7% (2/30) of all PRMT5 transcripts. In the pool of 30 analyzed clones, we did not detect any PRMT5 v6 splice variant. Based on these results, PRMT5 v1 and v2 were chosen for further analyses. We first assayed the effect of PRMT1, PRMT3, PRMT5 (v1 & v2) and MEP50 overexpression on HBV replication and transcription. HepG2_hNTCP cells were transfected with selected PRMTs expression plasmids and 48 h post-transfection infected with HBV (MOI 1000 viral genome equivalents [VGE]/cell). Four days after infection, HBV replication was evaluated by quantitative PCR (qPCR) using primers specific for total HBV DNA (Fig 1B) and cccDNA (Fig 1C). Overexpression of all tested PRMTs resulted in the reduction of the intracellular HBV DNA pool by approximately 40–60%. Interestingly, the decrease in HBV DNA was accompanied by the reduction of the level of cccDNA from 25 to 45%. To further evaluate the role of PRMTs in HBV replication, we tested the effect of the PRMTs’ overexpression on HBV transcription. The HBV transcription was evaluated four days post-infection by quantitative RT-PCR (RT-qPCR) using primers specific for HBV pgRNA and preC RNA (Fig 1D). The cccDNA transcription activity was determined as ratio of RNA to cccDNA. We found that overexpression of PRMT5v1 and v2 resulted in significant down-regulation of cccDNA transcription by approximately 50%. The levels of PRMTs’ overexpression were estimated by Western blot using anti-Flag antibodies (Fig 1E).

**Fig 2:**
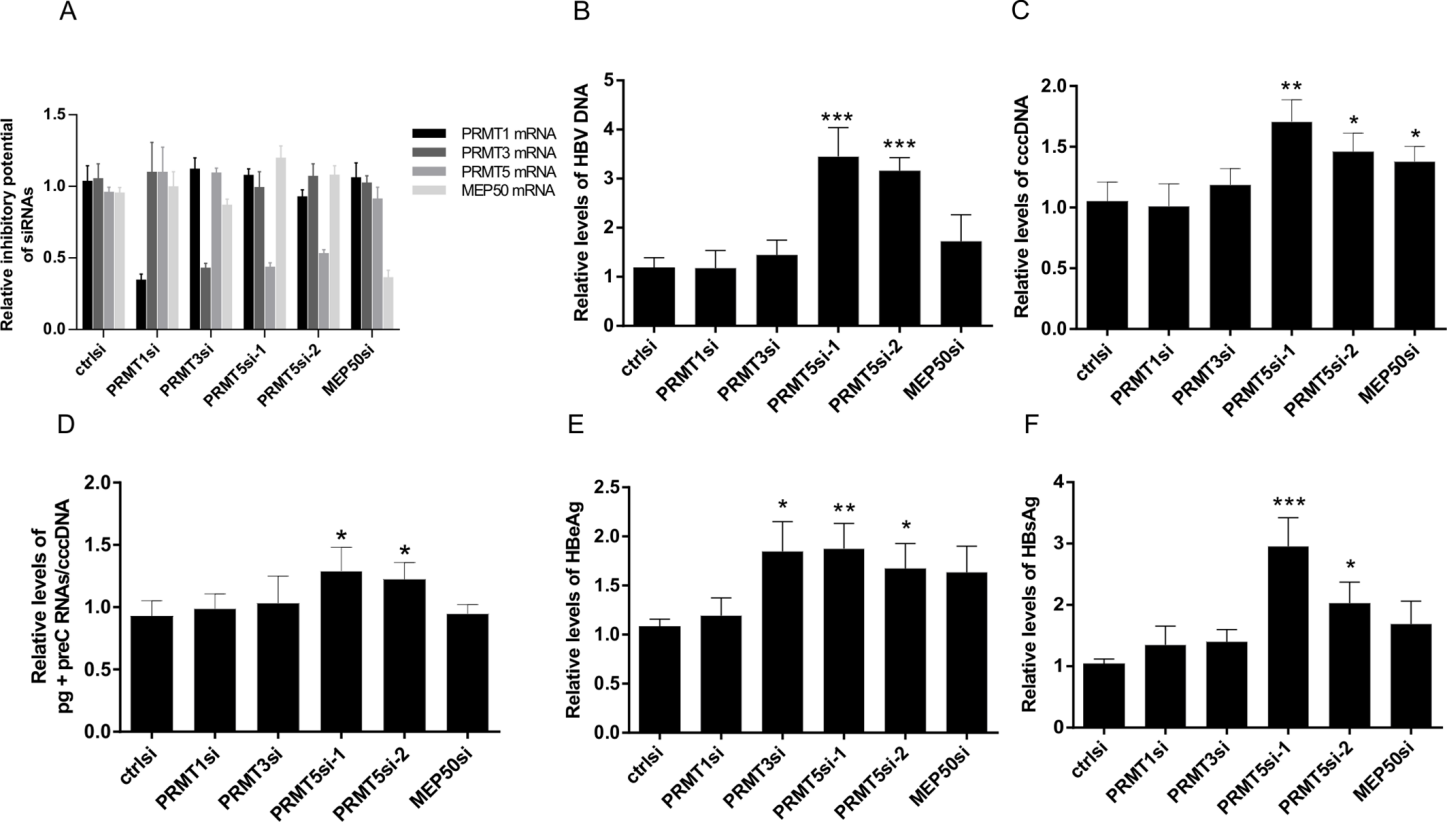
Inhibition of PRMT5 expression stimulates HBV replication and secretion of HBV antigens. (A) Down-regulation of endogenous PRMT1, 3, 5, and MEP50 mRNA expression in siRNA-transfected HepG2_hNTCP cells was estimated three days post-infection by RT-qPCR using primers specific for PRMT1, 3, 5, and MEP50 cDNAs and subsequently normalized to the expression of β-actin. Error bars represent SD of four independent experiments. (B) and (C) HepG2_hNTCP cells were transfected with specific siRNAs targeting PRMT1, PRMT3, PRMT5, MEP50, or non-targeting-ctrlsiRNA. Forty-eight hours after transfection, cells were infected with HBV (1000 VGE/cell). Four days post-infection, cells were harvested for DNA and RNA isolation. The levels of total HBV DNA (B) and cccDNA (C) were determined by qPCR and normalized to albumin. (D) HBV RNA (pgRNA+preC RNA) transcription was analyzed by RT-qPCR and normalized to cccDNA. HBeAg (E) and HBsAg (F) in the culture supernatants were analyzed by enzyme-linked immunosorbent assay (ELISA) four days post-infection. Asterisks indicate statistically significant differences between the control (ctrlsiRNA) and PRMTsiRNA groups determined by ANOVA (* P<0.05; ** P<0.01; *** P<0.001). Error bars represent SD of three independent experiments.

To further confirm the PRMTs’ inhibitory effect on HBV replication, we studied the HBV replication upon depletion of PRMTs, their splicing variants and MEP50. Endogenous PRMTs’ expression in HepG2_hNTCP cells was reduced using specific siRNAs. For down-regulation of PRMT5, we used two different siRNAs, 1 and 2, which were specific for exons 8 and 7, respectively (Fig 1A) and thus targeted all known PRMT5 splice variants (Fig 1A). The specificity and efficiency of siRNAs was tested using RT-qPCR approach and the results were summarized in Fig 2A. As shown in Fig 2B, down-regulation of PRMT5 expression resulted in significant ~ 3 (PRMT5siRNA-2) to 3.5 (PRMT5siRNA-1) -fold up-regulation of the total HBV DNA pool. These data correlated with an increase of cccDNA level (1.3- to 1.7-fold) in cells transfected with siRNAs specific for PRMT5 and MEP50 (Fig 2C). Although the transfection of PRMT1- and PRMT3-specific siRNAs resulted in the reduction of their respective mRNAs (Fig 2A), we did not detect a significant increase in both total HBV and cccDNA levels (Fig 2B and 2C). Since both PRMT1- and PRMT3- siRNAs were designed to target all known PRMT1 and PRMT3 splice variants, the failure to detect any effect on HBV replication could possibly be due to some compensatory mechanism, which activates a different enzyme within the family of type I methyltransferases that is involved in the same pathway. We also studied the PRMT-specific siRNA effect on HBV pgRNA and preC RNA expression. As shown in Fig 2D, a significant up-regulation of HBV RNA was detected only upon PRMT5 knock-down. In contrast, the knock-down of PRMT1 and PRMT3 and MEP50 had no effect on HBV transcription. Secretion of HBsAg and HBeAg into cell-free supernatant was analyzed at day 4 post-infection. We observed statistically significant increases in the levels of HBeAg in media of cells transfected with PRMT3- and PRMT5- siRNAs compared to the control (ctrlsiRNA) (Fig 2E). However, a significant increase in the levels of secreted HBsAg was detected only in the media of cells transfected with PRMT5siRNAs (2.9- and 2-fold increase compared to control group) (Fig 2F).

While, based on overexpression studies, all selected PRMTs appeared to have inhibitory potential on HBV replication, the data generated from the siRNA experiments led to the conclusion that only PRMT5 is a restriction factor of HBV replication. Therefore, our next analyses were primarily focused on PRMT5, its co-factor MEP50, and their interaction with HBV.

### HBc interacts with PRMT3, PRMT5, and MEP50

**Fig 3:**
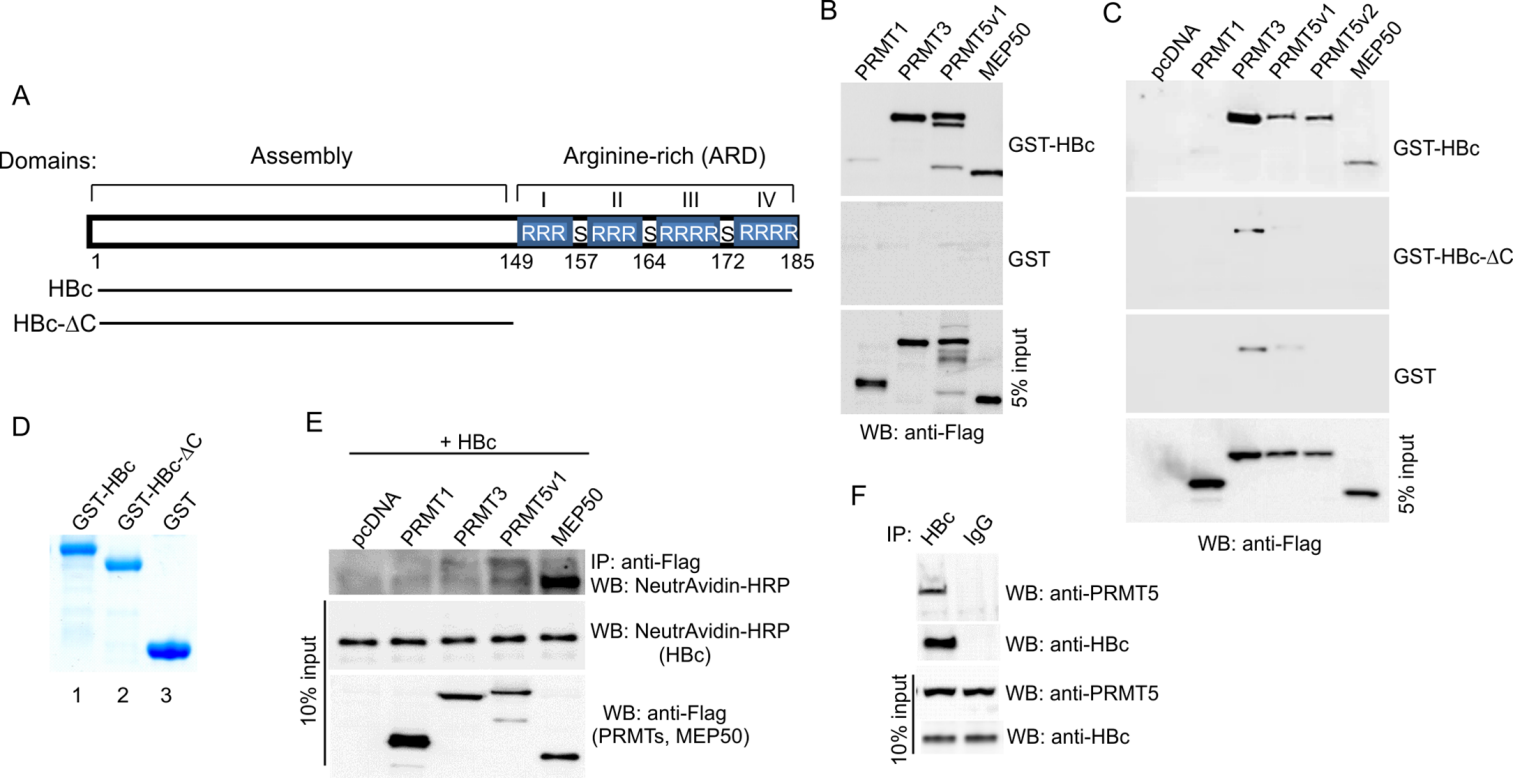
HBc protein interacts with PRMT3, PRMT5 and MEP50. (A) Schematic representation of HBc (Genotype A, subtype adw2) and its C-terminal deletion mutant (HBc-ΔC). ARD, Arginine-rich domain; S, serine residues modified by phosphorylation. (B) Flag-tagged PRMT1, PRMT3, PRMT5v1 and MEP50 were *in vitro* translated using TNT T7 quick coupled transcription/translation system and incubated with HBc fused to GST or GST alone immobilized on glutathione-Sepharose beads. The bound proteins were eluted and resolved on 10% SDS-PAGE followed by Western blot with anti-Flag antibodies. Five % of PRMTs´ protein input is shown below (5% input). (C) HBc protein interacts with PRMT3, PRMT5 and MEP50 via its C-terminal domain. HEK293T cells were transfected with equal amounts of Flag-tagged PRMT1, PRMT3, PRMT5 (v1, v2) and MEP50 expression constructs. Forty-eight hours after transfection, the cell lysates were incubated with HBc (aa 1–185), HBc-ΔC (aa 1–149) fused to GST or GST alone immobilized on glutathione-Sepharose beads. The bound proteins were eluted and resolved on 10% SDS-PAGE followed by Western blot with anti-Flag antibodies. Five % of PRMTs´ protein input is shown below (5% input). (D) Coomassie blue staining of purified GST, GST-HBc, and GST-HBc-ΔC used in GST pull-down experiments. (E) Co-immunoprecipitation of HBc and PRMTs in transfected HEK293T cells. HEK293T cells were transfected with Flag-tagged PRMTs’ expression plasmids and HBc-V5/AP expression plasmid, as indicated. Forty-eight hours after transfection, the cells were harvested and protein lysates were prepared. Protein lysates (400 μg) were immunoprecipitated (IP) with anti-Flag antibodies, and the immunoprecipitated complexes were analyzed by Western blot (WB) with NeutrAvidin conjugated to HRP. The relative levels of PRMTs and HBc in 40 μg of protein lysates are shown for comparison (10% input). (F) Co-immunoprecipitation of HBc and endogenous PRMT5 in HepG2.2.15 cells. Protein lysates (400 μg) isolated from HepG2.2.15 cells were immunoprecipitated (IP) with anti-HBc or control (IgG) antibodies. The immunoprecipitated complexes were analyzed by Western blot (WB) with anti-PRMT5 and anti-HBc antibodies. The relative levels of PRMT5 and HBc in 40 μg of protein lysates are shown in bottom panels (10% input).

Due to the pleiotropic nature of the HBc protein function and its involvement in all phases of HBV life cycle, we examined whether HBc interacts with PRMTs and MEP50. The full-length HBc protein contains two distinct domains connected by a hinge region. The N-terminus is an assembly domain of HBc 1–140 aa, and the C-terminus is the ARD of HBc 150–185 aa (Fig 3A). The ARD domain consists of four arginine clusters containing three to four arginine residues.

We first examined the interaction of HBc with PRMTs *in vitro* by GST pull-down assay and then *in vivo* by co-immunoprecipitation. The results in Fig 3B showed that *in vitro* translated PRMT3, PRMT5v1 and MEP50 bound strongly to the full-length GST-HBc fusion protein, whereas, they did not interact with GST alone. We did not detect any interaction between PRMT1 and HBc. Notably, *in vitro* translation of PRMT5v1 yielded three distinct bands of different molecular weights probably due to multiple translation events originating from downstream ATGs. Interaction between HBc and PRMT3, 5 and MEP50 was confirmed by GST pull-down assay using cell lysates prepared from transfected HEK293T cells (Fig 3C). To determine whether the ARD region of HBc is required for interaction with PRMTs, we generated a GST-HBc-ΔC deletion mutant (1–149 aa). HEK293T cells were transfected with Flag-tagged PRMTs and MEP50 and cell lysates were incubated with the full-length GST-HBc, GST-HBc-ΔC or GST alone. The quality of purified recombinant GST-HBc, GST-HBc-ΔC and GST proteins is shown in Fig 3D (lanes 1, 2 and 3, respectively). The GST pull-down analysis revealed that ARD of HBc (amino acid residues 150–185) was important for the interaction with PRMT3, PRMT5 (v1 and v2) and MEP50. The *in vivo* interaction between ectopically expressed HBc and PRMTs in HEK293T cells was further examined by co-immunoprecipitation. The HBc-V5/AP protein modified by AP tag, that allows intracellular biotinylation, co-precipitated with Flag-tagged PRMT5 and MEP50 (Fig 3E). Interestingly, the HBc protein, which co-precipitated with PRMT5, displayed two sizes, one corresponding to *wt*HBc protein and the second one approximately 8–10 kDa larger, which may represent a post-translationally modified form of the HBc protein. We also detected a weak interaction between HBc and PRMT3, but there was no detectable association between PRMT1 and HBc protein. The interaction between HBc and endogenous PRMT5 was also confirmed by co-immunoprecipitation in HBV-transfected HepG2.2.15 cells (Fig 3F). Collectively, this data suggests that HBc interacts with both PRMT5 and MEP50 via its C-terminally located arginine-rich domain.

### HBc is methylated on arginine residues located at the carboxyl-terminus

**Fig 4:**
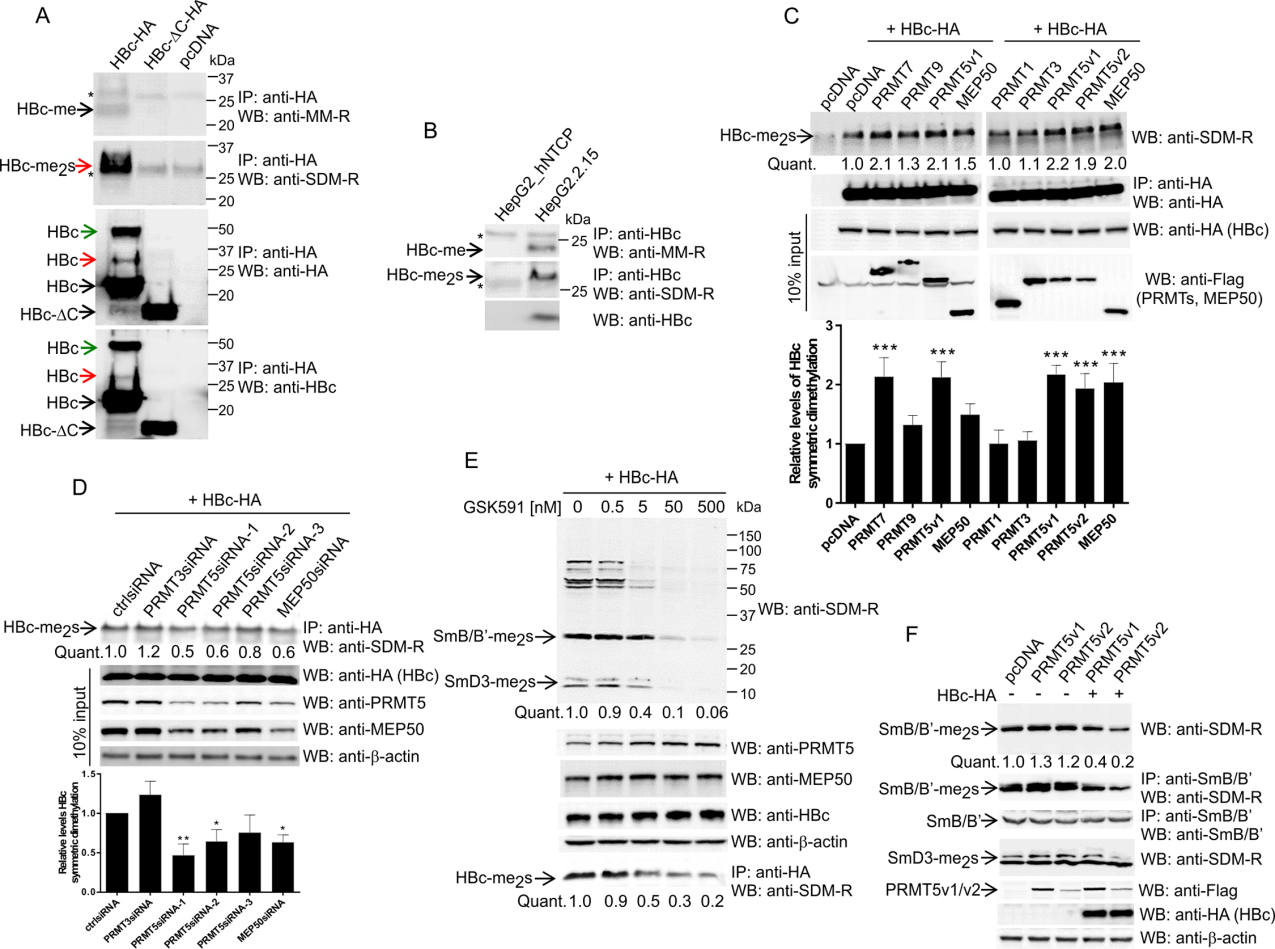
Symmetric dimethylation and monomethylation of arginine residues at the C-terminal domain of HBc protein. (A) Detection of monomethylation and symmetric dimethylation of HBc protein. HepG2_hNTCP cells were transfected with HBc-HA (full length, aa 1–185), HBc-ΔC-HA (C-terminal deletion, aa 1–149), or an empty vector (pcDNA). Forty-eight hours after transfection, cell lysates (400 μg) were precipitated with anti-HA antibodies (HBc and HBc-ΔC), and the precipitated complexes were analyzed by Western blot with monomethyl-arginine (MM-R, top panel) or symmetric-dimethyl-arginine (SDM-R, middle panel) antibodies. The quality/efficiency of anti-HA immunoprecipitation is shown by Western blot with anti-HA or anti-HBc antibodies in the bottom panels. *, non-specific WB signal. Red and green arrows show HBc protein fragments of 30 kDa and 48 kDa, respectively. (B) Cell lysates (400 μg) of HepG2.2.15 cells were precipitated with HBc antibodies and the precipitated complexes were analyzed by Western blot with monomethyl-arginine (MM-R, top panel) or symmetric-dimethyl-arginine (SDM-R, middle panel) antibodies. The expression level of HBc protein is shown in the bottom panel. *, non-specific. (C) Symmetric dimethylation of HBc is stimulated by PRMT5, PRMT7 and MEP50. HEK293T cells were co-transfected with a constant amount of HBc-HA expression plasmid in combination with expression plasmids encoding Flag-tagged PRMT1, PRMT3, PRMT5 (v1, v2), PRMT7, PRMT9, MEP50, or an empty vector. Forty-eight hours after transfection, cell lysates (400 μg) were precipitated with anti-HA antibodies (HBc), and the precipitated complexes were analyzed by Western blot with SDM-R antibodies. The relative levels of transfected HBc-HA and Flag-PRMTs or Flag-MEP50 in 40 μg of cell lysates were estimated by Western blots (10% input). The intensity of the symmetric dimethylation signal was quantified using the ImageQuant TL Array software and normalized to the level of HBc expression. The level of HBc methylation in mock (pcDNA) transfected cells was set to 1 (Quant. and graph below). Asterisks indicate statistically significant differences between the control (pcDNA) and PRMTs groups determined by ANOVA (*** P<0.001). Error bars represent SD of three independent experiments. (D) Down-regulation of PRMT5 and MEP50 inhibits symmetric dimethylation of HBc protein. HEK293T cells were co-transfected with a constant amount of HBc-HA expression plasmid in combination with PRMT3-, PRMT5- or MEP50-specific siRNAs, or control siRNA (ctrlsiRNA). Forty-eight hours after transfection, cell lysates (400 μg) were precipitated with anti-HA antibodies (HBc), and the precipitated complexes were analyzed by Western blot with SDM-R antibodies. The relative levels of endogenous PRMT5, MEP50, β-actin or transfected HBc-HA in 40 μg of cell lysates were estimated by Western blots (10% input). The intensity of the symmetric dimethylation signal was quantified as described in (C). The level of HBc methylation in mock (ctrlsiRNA) transfected cells was set to 1 (Quant. and graph below). Asterisks indicate statistically significant differences between the control (ctrlsiRNA) and PRMTsiRNA groups determined by ANOVA (* P<0.05; ** P<0.01). Error bars represent SD of three independent experiments. (E) Treatment with GSK591, a potent PRMT5/MEP50-specific inhibitor, reduces the symmetric arginine dimethylation of HBc protein. HepG2_hNTCP cells were transfected with equal amounts of HBc-HA expression plasmid. Twenty-four hours after transfection, the cells were treated with increasing concentrations of GSK591 (0, 0.5, 5, 50, 500 nM) for 72 hours as indicated. The cell lysates (40 μg) were analyzed by Western blotting (WB) with SDM-R, PRMT5, MEP50, HBc and β-actin antibodies. To analyze the level of symmetric-arginine dimethylation of HBc protein (bottom panel), the cell lysates (400 μg) were immunoprecipitated (IP) with anti-HA-specific antibodies followed by Western blotting with anti-SDM-R antibodies. The intensities of total cellular symmetric dimethylation and HBc protein dimethylation were quantified as described in (C) and normalized to the levels of β-actin or HBc expression, respectively. (F) Expression of HBc inhibits the PRMT5-mediated methylation of SmB/B’ and SmD3 splicing factors. HEK293T cells were co-transfected with a constant amount of HBc-HA expression plasmid in combination with expression plasmids encoding Flag-tagged PRMT5v1 and v2, or an empty vector, pcDNA, as indicated. Forty-eight hours after transfection, cell lysates (20 μg) were analyzed by Western blot with SDM-R, β-actin, Flag and HA antibodies. The levels of symmetrically dimethylated SmB/B’and SmD3 were quantified as described in (C) and normalized to the levels of β-actin expression. To confirm that the major methylated band corresponds to SmB/B’, the SmB/B’protein was immunoprecipitated from cell lysates (400 μg) with anti-SmB antibodies followed by Western blotting with anti-SDM-R antibodies (middle panels).

We next investigated whether HBc protein can be methylated in cells. Since we showed that HBc associates with type II enzyme PRMT5 and its co-factor MEP50, we focused on arginine monomethylation and symmetric dimethylation. HepG2_hNTCP cells were transfected with HA-tagged full-length and ΔC deletion mutant of HBc protein. Forty-eight hours after transfection, HBc protein was precipitated from cell lysates using anti-HA magnetic beads and analyzed by Western blot with specific anti-Mono-Methyl-Arginine (MM-R) and anti-Symmetric-Di-Methyl-Arginine (SDM-R) antibodies. As shown in Fig 4A (upper panel), MM-R antibodies detected band of 22 kDa (black arrow) which is identical with predicted size of HBc-HA protein. Western blot with SDM-R antibodies (Fig 4A, middle panel) detected a single band of approximately 30 kDa, which is rather larger than expected size of HBc-HA. An 8–9 kDa increase in molecular mass could be due to modification by methyl groups together with other types of post-translational modification, e.g. serine phosphorylation or monoubiquitination. Interestingly, two high molecular weight bands migrating at 30 kDa and 48 kDa were also detected when the blot was re-probed with anti-HA or anti-HBc antibodies [Fig 4A, bottom panels, red (30 kDa), green (48 kDa) arrows], suggesting that the detected fragments originated from HBc protein and are not due to co-precipitation of some cellular proteins. Notably, neither monomethylation, nor symmetric dimethylation was detected using the HBc-ΔC deletion mutant. This data demonstrated that HBc protein is modified by methylation on arginine residues at the C-terminal part. This type of modification may be further linked to other types of PTMs, e.g. phosphorylation or ubiquitination.

To study the HBc methylation in the context of the whole HBV genome and viral protein expression, the HBc protein was immunoprecipitated from HepG2.2.15 cell lysates and analyzed by immune blotting with MM-R and SDM-R antibodies. In agreement with previous experiment, Western blot with MM-R and SDM-R antibodies yielded bands with molecular mass of approximately 22 and 30 kDa, respectively (Fig 4B). The HBc protein expressed in HepG2.2.15 cells represents subtype ayw and consists of 183 amino acids. Both HBc proteins, adw2 (185 aa; used in this study) and ayw (183 aa; expressed in HepG2.2.15), are almost identical in their ARD domains, except a two amino acid (DR) insertion in adw2 HBc at position 153–154.

It was important to establish which PRMT is responsible for methylation of HBc in cells. Therefore, we included in our analyses also PRMT7 and PRMT9. PRMT7 has been shown to display weak type II activity, but it is primarily responsible for depositing the MMA mark, thus categorizing it as a type III enzyme [36]. PRMT9 was recently described as type II methyltransferase that methylates the splicing factor SAP145 [29]. To analyze the arginine methylation of HBc, we co-expressed HBc-HA with PRMT5 (v1, v2), PRMT1, 3, 7, 9 and MEP50 and thereafter assessed the level of symmetrically dimethylated HBc (Fig 4C). Methylated HBc was readily detected upon anti-HA immunoprecipitation, and the increase in level of methylation, determined by densitometric tracing of Western blot, was approximately 2-fold after co-transfection of PRMT5, PRMT7 and MEP50 when compared to mock-transfected control (Fig 4C, line Quant. and graph below). As expected, symmetric dimethylation of HBc was not affected by PRMT1 and PRMT3 and was only slightly increased (1.3 fold) by PRMT9. To confirm the involvement of PRMT5-MEP50 in control of HBc methylation, we also assessed HBc methylation status upon depletion of PRMT5 and MEP50 from cells by means of RNA interference. As shown in Fig 4D, transfection of three PRMT5-specific siRNAs (the positions of targeted sequences are depicted in Fig 1A), and one MEP50-specific siRNA resulted in the reduction of endogenous PRMT5 and MEP50 in cells. Interestingly, PRMT5-specific siRNAs reduced also the levels of MEP50 protein and conversely, siRNA targeted against MEP50 down-regulated also the levels of PRMT5. These results indicate that the stability of PRMT5 and MEP50 proteins is co-regulated. As apparent from Fig 4D, the depletion of both PRMT5 and MEP50 led to decreased levels of symmetrically dimethylated HBc, the relative methylation level of HBc changed from 1.0 in control siRNA-transfected cells to 0.8–0.5 in PRMT5siRNA- and MEP50siRNA-transfected cells (Fig 4D, line Quant. and graph below). As expected, the PRMT3-specific siRNA did not decrease the level of symmetrically dimethylated HBc. To further confirm the role of PRMT5 in HBc methylation, we also assayed the symmetric dimethylation of HBc protein in the presence of the PRMT5-specific inhibitor EPZ015866 (GSK591) (Fig 4E). GSK591 potently inhibits the PRMT5/MEP50 complex from methylating histone 4 (H4) (IC_50_ = 11 nM) and SmD3 (EC_50_ = 56 nM). Furthermore, GSK591 is selective for PRMT5 (up to 50 micromolar) relative to a panel of methyltransferases [37, 38]. To determine the effect of GSK591 treatment on symmetric arginine dimethylation of HBc and cellular proteins in HepG2_hNTCP cells, the cells were transfected with constant amounts of HBc-HA and 24 h post-transfection the cells were treated with increasing concentrations (0, 0.5, 5, 50, 500 nM) of the GSK591 compound for a total of 72 hrs (Fig 4E). The cellular symmetric dimethylation was assayed by Western blot with SDM-R antibodies. The identity and characterization of symmetrically dimethylated cellular proteins was studied previously [39, 40]. In general, antibodies that are specific to symmetrically dimethylated arginine residues recognize endogenous levels of methylated SmB/B’ (~30 kDa), SmD (D1/D2/D3, ~16 kDa) and p80-Coilin (~80 kDa). As shown in Fig 4E, the GSK591 treatment led to a concentration-dependent decrease in the intensity of methylation of multiple cellular proteins including Sm proteins (SmB/B’ and SmD3). Concentration-dependent decrease in symmetric dimethylation was also observed for HBc protein with 0.5-fold reduction in methylation at compound concentration of 5 nM. Collectively, this data indicated that PRMT5 is involved in the methylation of HBc.

To determine, if HBc protein could compete with other cellular substrates for binding to PRMT5-MEP50 complex and thus inhibit their methylation, we assayed the methylation level of two spliceosomal Sm proteins, SmB/B’ and SmD3, in the presence of HBc. As shown in Fig 4F, while overexpression of PRMT5 (v1, v2) moderately increased the methylation of SmB/B’and SmD3 proteins (1.2- and 1.3-fold for PRMT5v2 and PRMT5v1, respectively; estimated by Quant. analysis), co-expression of PRMT5 and HBc resulted in significant down-regulation of methylated SmB/B’ and SmD3 (0.2- and 0.4-fold for HBc-HA co-transfected with PRMT5v2 and PRTM5v1, respectively; estimated by Quant. analysis). The effect of HBc protein on methylation of SmB/B’was also studied on immunoprecipitated SmB/B’protein (Fig 4F, middle panels). While the levels of endogenous SmB/B’ remained constant, their arginine dimethylation was clearly reduced in the presence of HBc protein. Hence, the binding of HBc to PRMT5-MEP50 may lead to decreased availability of this complex and subsequently reduced methylation of cellular substrates.

### Analysis of mono and dimethylation of HBc protein

**Fig 5:**
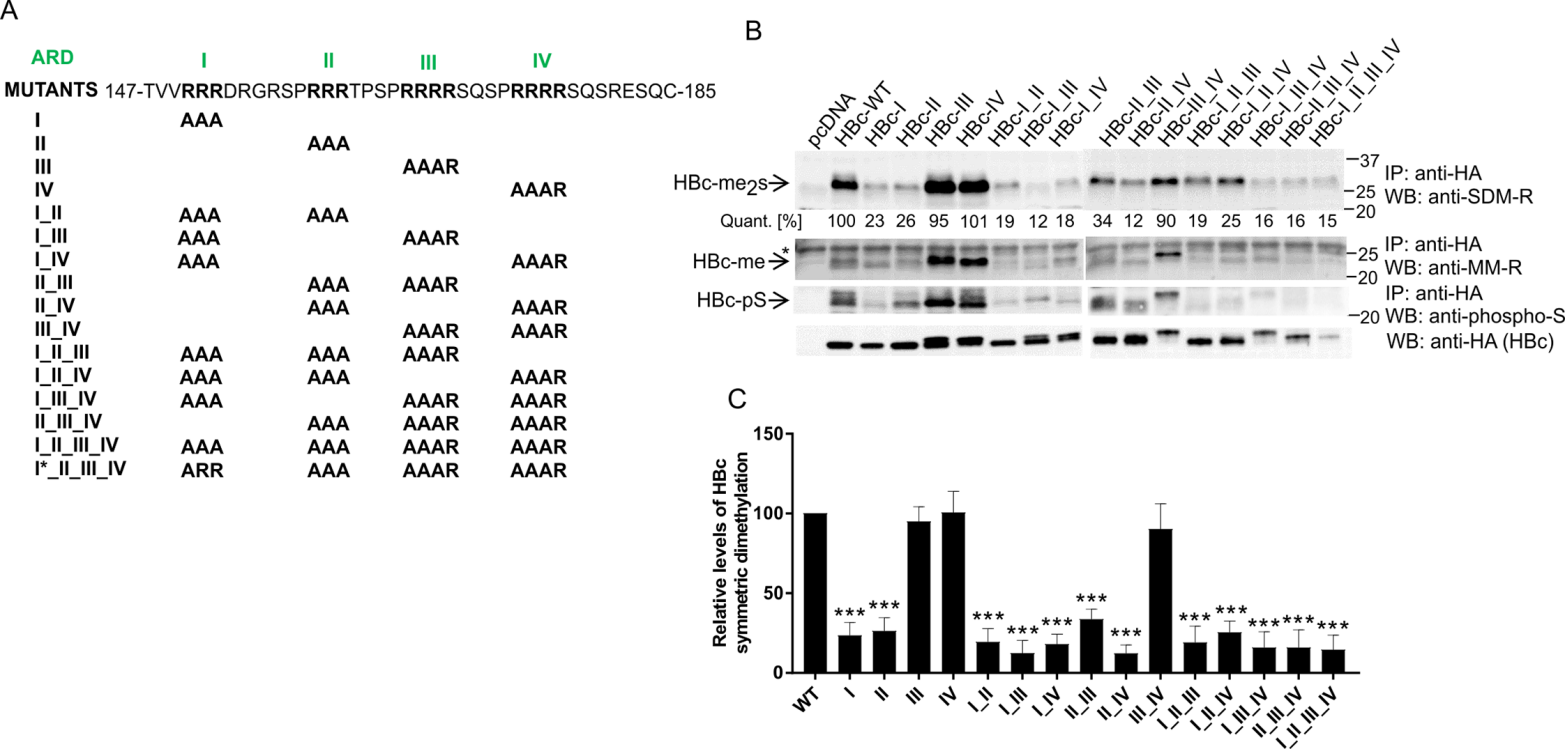
Analysis of arginine methylation of HBc ARD mutants. (A) Schematic representation of 16 arginine-to-alanine mutations in arginine-rich-domain (ARD) of HBc protein. The sequence of *wild type* HBc ARD corresponds to HBV genotype A, subtype adw2. (B) Analysis of monomethylation, symmetric dimethylation and serine phosphorylation of HBc *wild type* (*wt*) and ARD mutants. HepG2_hNTCP cells were transfected with equal amounts of HA-tagged HBc *wt* and ARD mutants. Forty-eight hours after transfection, cell lysates (400 μg) were precipitated with anti-HA antibodies (HBc), and the precipitated complexes were analyzed by Western blot with SDM-R, MM-R and phospho-serine antibodies. The relative levels of transfected HBc-HA in 40 μg of cell lysates were estimated by Western blots (bottom panel). The intensity of symmetric dimethylation signal was quantified using ImageQuant TL Array software and normalized to the level of HBc expression. The values were calculated as means of three independent transfection experiments. *, non-specific WB signal. (C) Graphical quantitation of the immunoblot signals from panel B. Levels of symmetric dimethylation were normalized to HBc expression. Asterisks indicate statistically significant differences between the control (wtHBc) and HBc ARD mutants determined by ANOVA (*** P<0.001). Error bars represent standard errors from three independent transfection experiments.

To map the HBc arginine methylation domain, we introduced arginine-to-alanine (R-to-A) substitutions at various positions of HBc ARD. A total of 16 HBc mutants were generated (Fig 5A). For each ARD domain (I to IV), three adjacent arginine residues were mutated to alanines, except for mutant I*_II_III_IV, which represented a cloning intermediate and was only included in Mass spectrometry analyses. A total of 15 ARD mutants and one *wt*HBc tagged with HA, were transfected into HepG2_hNTCP cells, prepared cellular lysates were immunoprecipitated with anti-HA antibody and analyzed by Western blot with anti-SDM-R and MM-R antibodies (Fig 5B). Upon quantification of the signal corresponding to symmetrically dimethylated HBc (HBc-me_2_s) and its normalization to HBc expression levels (Fig 5B, bottom panel and Fig 5C), we concluded that the highest level of methylation was detected in *wt*HBc (100%) and ARD mutants which carried the *wt* status of both ARDI and ARDII. This meant that mutation of either ARDI or ARDII significantly decreased the degree of symmetric dimethylation (Fig 5C). Likewise, the HBc monomethylation displayed almost identical pattern (Fig 5B). Since C-terminal phosphorylation of HBc protein was shown to be critical for generating viral particles that are fully capable of viral replication [8, 41], we also analyzed the levels of serine phosphorylation of *wt*HBc and ARD mutants (Fig 5B). Again, similarly to arginine methylation, the highest levels of phosphorylation were displayed by *wt*HBc as well as ARD mutants with intact ARDI and II domains.

Based on the presented data, we postulated the following conclusions: i) an efficient HBc methylation requires the presence of intact ARDI and ARDII domains. ii) methylation probably occurs on arginine residues residing in these two domains or their immediate vicinity. However, some residual methylation may possibly occur also outside this region. iii) due to the fact that analysis of symmetrically dimethylated HBc yielded band with considerable higher molecular weight, the symmetrical dimethylation may be linked to other types of PTMs, e.g. phosphorylation, ubiquitination, etc.

### Mass spectrometry analysis of HBc identified multiple potential ubiquitination, phosphorylation and arginine methylation sites

**Fig 6:**
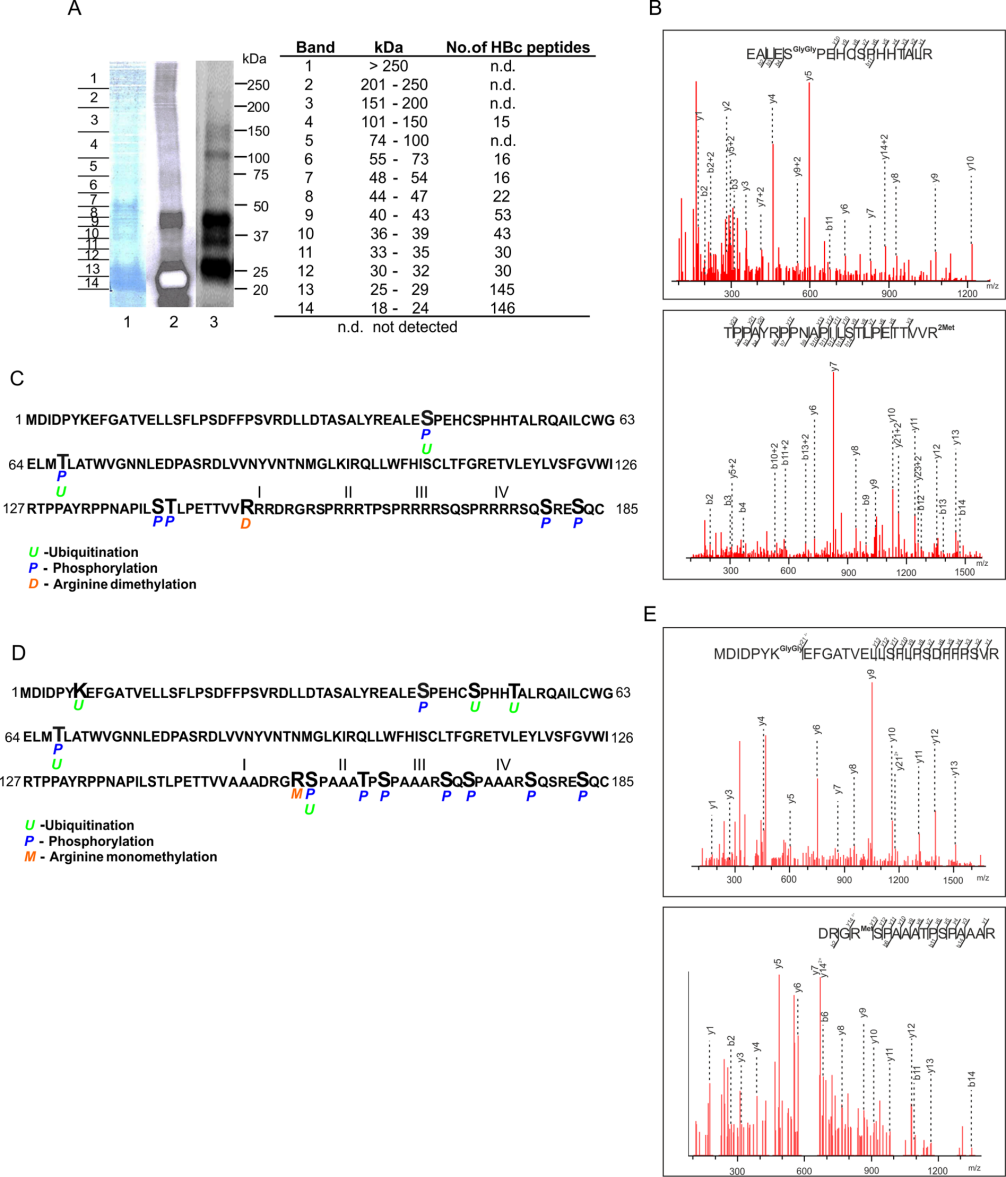
Mass spectrometry analysis of HBc and its PTMs. (A) MS analysis detected high molecular structures of HBc protein. Protein lysates isolated from HBc-HA transfected HepG2_hNTCP cells were subjected to immunoprecipitation with anti-HA magnetic beads. The immunoprecipitated HBc protein was resolved by SDS-PAGE and stained by Coomassie blue (lane 1), visualized by Western blot with anti-HBc (lane 2) or anti-Ub (lane 3) antibodies. The Coomassie stained gel was cut into 14 slices and resulting bands were analyzed by LC-MS/MS. The column “No. of HBc peptides” corresponds to number of peptides spectra identified with confidence higher than 95% as assigned by ProteinPilot. (B) Tandem mass spectra of peptides derived from *wt*HBc showing ubiquitin conjugation with di-Gly modification (upper panel) of specific HBc residues. The lower panel represents the spectrum for dimethyl modification of *wt*HBc at R150. (C) MS analysis of immunoprecipitated *wt*HBc revealed multiple ubiquitinated (*U*), arginine dimethylated (*D*) and phosphorylated (*P*) sites. I, II, III, IV–four arginine-rich domains. (D) Potential PTMs of HBc detected by MS analysis of HBc mutant proteins (I, I_II_III, I_II_IV, II_III_IV, I_II_III_IV and I*_II_III_IV). The amino acid sequence is derived from HBc mutant I_II_III_IV containing mutations of all four arginine-rich clusters. *U*, ubiquitination; *P*, phosphorylation; *M*, arginine-monomethylation. (E) Tandem mass spectra of peptides derived from HBc mutant (I_II_III) showing ubiquitin conjugation with di-Gly modification at K7 (upper panel). The lower panel represents spectrum of monomethyl modification of HBc mutant (I*_II_III_IV) at R156.

Our data suggested that HBc protein could form several proteoforms due to multiple covalent post-translational modifications, including ubiquitination that can result in high molecular mass structures. To identify as many modifications as possible, we immunoprecipitated HBc protein from transfected HepG2_hNTCP cells and subjected it to SDS-PAGE. The gel was cut into fourteen slices (Fig 6A) and subjected to in-gel tryptic digestion. Peptides were extracted and analyzed by LC-MS/MS. In addition to in-gel approach, we also analyzed peptides bearing PTMs in samples digested directly on anti-HA beads. Database searching of gel experiment resulted in the identification of HBc-specific peptides distributed in gel slices 4 through 14 (except slice 5) which corresponded to molecular mass between 18 to 150 kDa (with exception of 74–100 kDa). As expected, the highest number of HBc-specific peptides was identified in gel slices corresponding to lower molecular weight of 18 to 29 kDa (altogether 291 peptides) and the region between 36 to 47 kDa (altogether 118 peptides). The bead experiment resulted in overall similar sequence coverage with overlaying evidence of PTMs. Thus MS analysis of *wt*HBc revealed identity of the ubiquitin peptides, with a GlyGly modification on serine (S44) (Fig 6B, upper panel) and threonine (T67) residues (Fig 6C). Both these residues appeared to be also phosphorylated. Similarly, we identified dimethylation on arginine R150 (Fig 6B, lower panel) and additional phosphorylation on serine (S141, S180, S183) and threonine (T142) residues (Fig 6C; data are available via ProteomeXchange with identifier PXD006828).

In both experiments we experienced insufficient coverage of the C-terminal part of the *wt*HBc sequence. The HBc ARD contains a total of 17 arginine residues (16 arginines in HBc of ayw serotype) that contribute to positive charge and render it difficult to be analyzed by applied experimental set-up (trypsin digestion produces short positively charged peptides that do not bind to the pre-column during desalting step). Therefore, HBc ARD mutants (I, I_II_III, I_II_IV, II_III_IV, I*_II_III_IV and I_II_III_IV) were also included in MS analyses. This experimental approach allowed coverage of 10 arginine residues with confidence above 95% and resulted in identification of additional PTMs, including ubiquitination at K7, S49, T53 and S157, arginine monomethylation at R156 and extensive phosphorylation of C-terminal residues (S157, T162, S164, S170, S172 and S178) (Fig 6D; data are available via ProteomeXchange with identifier PXD006828). The representative tandem mass spectra demonstrating ubiquitin-like modification at K7 and monomethylation at R156 are shown in Fig 6E). Collectively, we identified multiple potential ubiquitin- methyl- and phosphoryl-modified HBc residues. Notably, the majority of phoshorylated residues (e.g. S157, S164, S170, S172, S178, T162) identified in this study, was described previously and their role in HBV replication, pgRNA encapsidation or HBc intracellular trafficking was determined [8, 9, 41–43].

### HBc is ubiquitinated on K7 and methylated on R150 and R156

**Fig 7:**
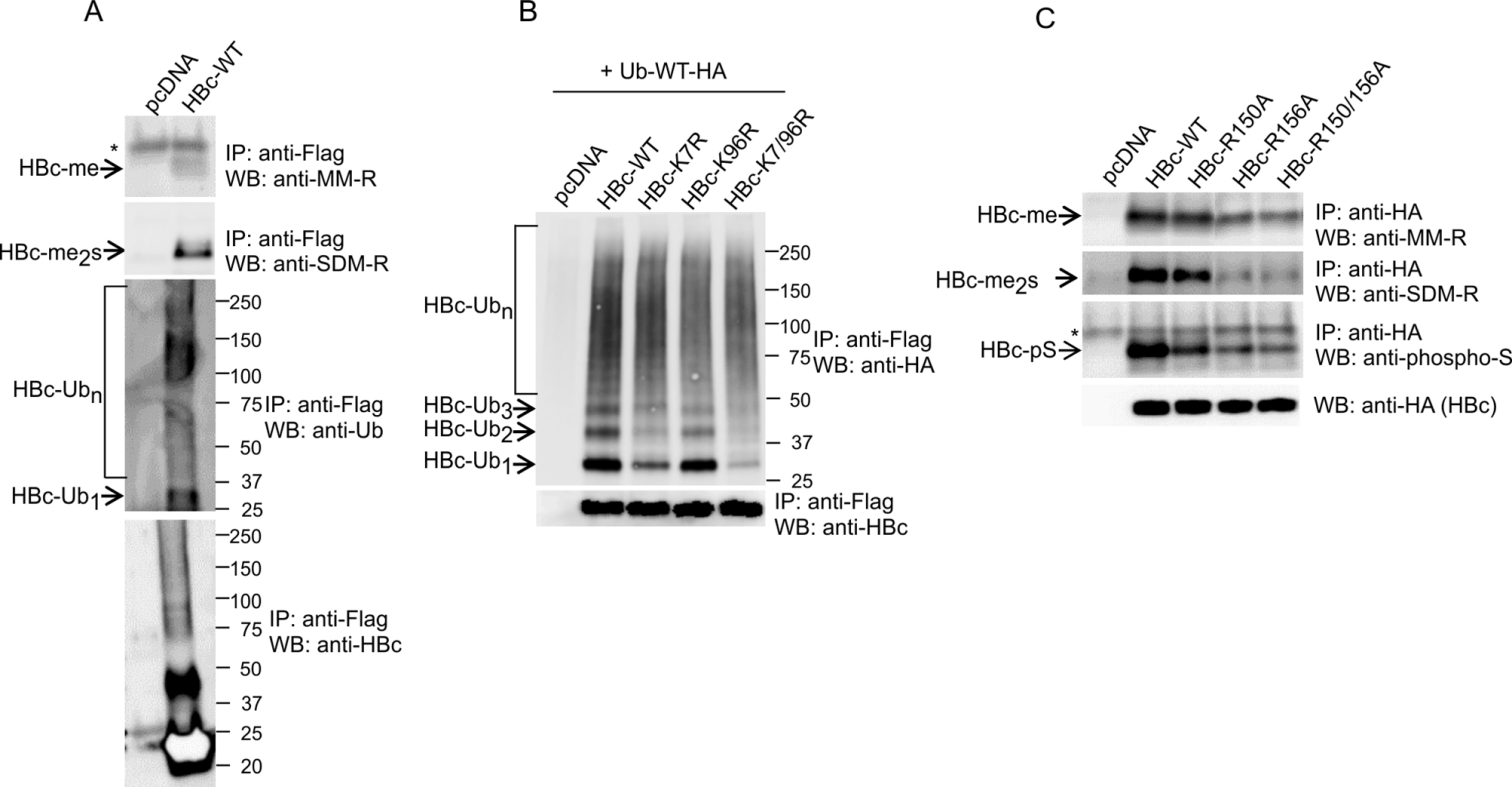
HBc protein is modified by ubiquitination on K7 and by methylation on R150 and R156. (A) HepG2_hNTCP cells were transfected with *wt*HBc tagged with Flag or an empty vector, pcDNA. Forty-eight hours after transfection, cell lysates (400 μg) were precipitated with anti-Flag antibodies (HBc), and the precipitated complexes were analyzed by Western blot with SDM-R, MM-R and Ub antibodies. The quality/efficiency of anti-Flag immunoprecipitation is shown in the bottom panel. (B) HepG2_hNTCP cells were transfected with *wt*Ub-HA together with *wt*HBc or K-to-R single and double mutants (HBc-K7R, HBc-K96R and HBc-K7/96R) tagged with Flag, as indicated. Forty-eight hours after transfection, cell lysates (400 μg) were precipitated with anti-Flag antibodies (HBc), and the precipitated complexes were analyzed by Western blot with Ub antibodies. The quality/efficiency of anti-Flag immunoprecipitation is shown in the bottom panel. (C) HepG2_hNTCP cells were transfected with *wt*HBc or R-to-A single and double mutants (HBc-R150A, HBc-R156A and HBc-R150/156A) tagged with HA, as indicated. Forty-eight hours after transfection, cell lysates (400 μg) were precipitated with anti-HA antibodies (HBc), and the precipitated complexes were analyzed by Western blot with SDM-R, MM-R and phospho-serine antibodies. The relative levels of transfected HBc-HA in 40 μg of cell lysates were estimated by Western blots (bottom panel). *, non-specific WB signal.

To address the possibility that HBc protein is modified by ubiquitination, we transfected *wt* HBc into HepG2_hNTCP cells and analyzed both HBc ubiquitination and arginine methylation. To rule out the possibility of detecting ubiquitinated cellular proteins that interact with HBc, we lysed the cells under stringent conditions of denaturing buffer (see Material and Methods). Under these conditions we showed that HBc protein can be mono-and poly-ubiquitinated as well as methylated (Fig 7A). Interestingly, the monoubiquitinated HBc migrated at the same position as symmetrically dimethylated HBc with approximate molecular mass of 30 kDa.

In most cases ubiquitination occurs on lysine residues. Ubiquitination on non-lysine residues (e.g. serines, threonines or cysteines) might reflect the ability of cell to ubiquitinate also proteins whose lysine residues are not exposed or lacking [44]. Since the HBc protein contains only two lysine residues, at positions 7 and 96, which are conserved across various HBV strains, we decided to study their potential involvement in ubiquitination. We mutated each individual lysine into arginine and analyzed their level of ubiquitination upon transfection into HepG2_hNTCP cells (Fig 7B). Mutation of K7 reduced the mono- and polyubiquitination of HBc protein. Interestingly, mutation of K96 affected the level of ubiquitination only marginally. Therefore, we concluded that HBc may be modified by ubiquitination at K7. Nevertheless, K7 is probably not the only residue that is ubiquitinated. As suggested by MS analysis, other non-canonical residues, like serines and threonines, may also be modified by ubiquitination and their evaluation awaits further studies.

To study the role of arginine residues, R150 and R156, in mono- and dimethylation, we prepared single and double R-to-A mutants of HBc. *Wild-type* and mutant HBc constructs were transfected into hepatocytic cell line, HepG2_hNTCP, immunoprecipitated using anti-HA antibodies and analyzed by Western blot with SDM-R and MM-R antibodies. As shown in Fig 7C, while monomethylation predominantly occurred at R156 (residue R154 for ayw HBc), symmetric dimethylation was detected on both R150 and R156 (residues R150 & R154 for ayw HBc). Notably, mutation of either arginine residue considerably compromised the level of HBc serine phosphorylation. This data suggests that symmetric dimethylation and serine phosphorylation are modifications that are linked or co-dependent.

### Role of ARDs in HBc subcellular distribution

**Fig 8:**
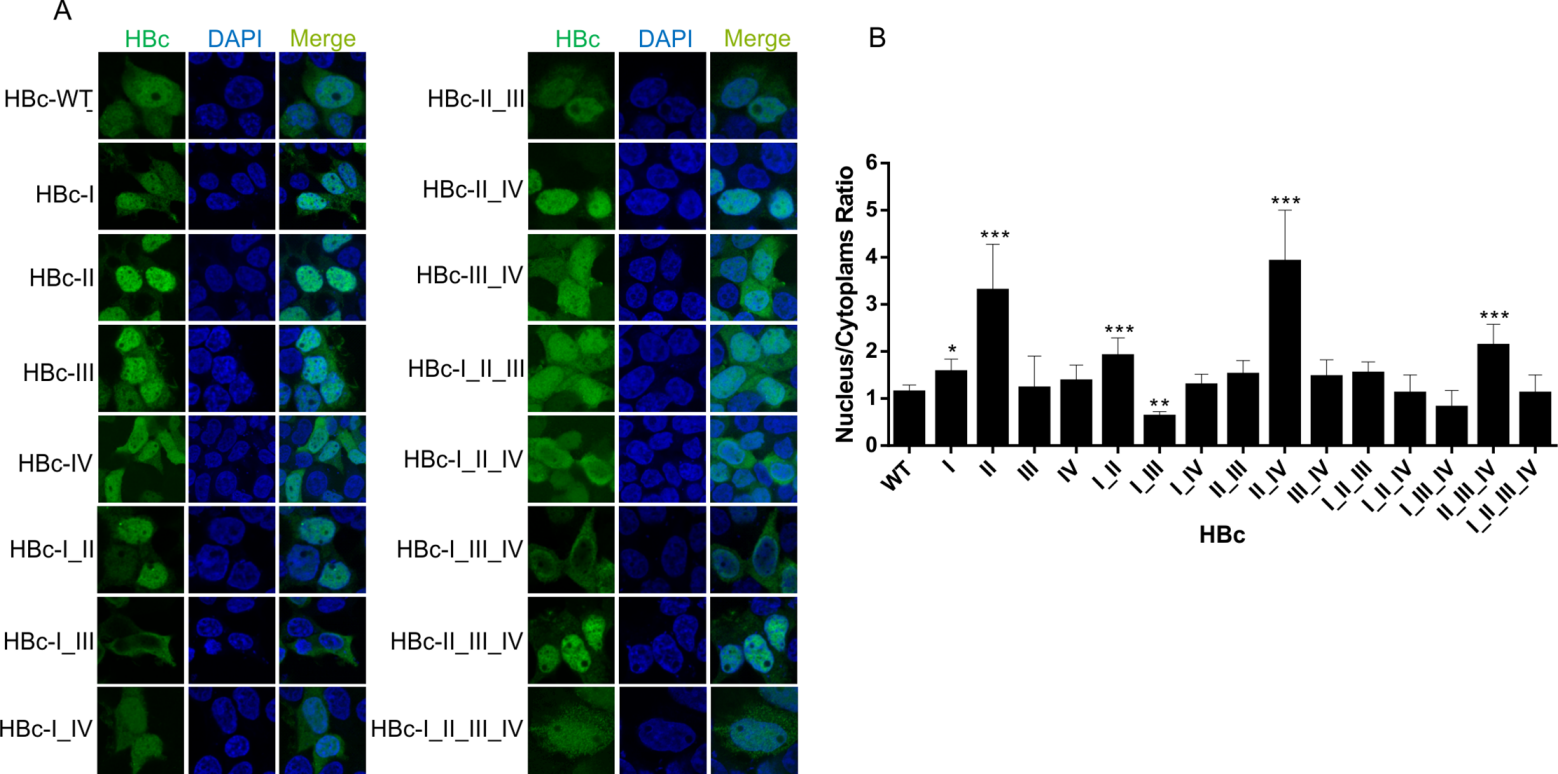
Subcellular distribution of HBc ARD mutants evaluated by immunofluorescence assay (IFA) in HEK293T cells. (A) Representative confocal microscopy images of the HA-tagged *wt*HBc and HBc ARD mutants visualized by FITC-conjugated anti-HA antibody in transfected HEK293T cells. HBc (green) and DAPI (blue). (B) Graphical analysis of single cell quantification experiments showing nuclear-to-cytoplasmic ratio of the average signal intensities measured for *wt*HBc and HBc ARD mutants. Error bars represent standard deviations (SD) calculated from the results of two independent transfection experiments involving at least 10-single cell analyses. Asterisks indicate statistically significant differences between the control (*wt*HBc) and the respective ARD mutants determined by ANOVA (* P<0.05; ** P<0.01; *** P<0.001).

The HBc ARD domains were previously implicated in control of nucleus-to-cytoplasm shuttling [13]. ARDI and ARDIII were shown to be associated with two co-dependent nuclear localization signals (NLS), while ARDII and ARDIV behave like two independent nuclear export (NES) or cytoplasm retention (CRS) signals [13]. Since these conclusions were based on a study of HBc ARD mutants with only two arginine residues mutated to alanines, we sought to determine whether the introduction of an additional mutation in our ARD mutants, would modify the subcellular distribution. The subcellular localization of HBc *wt* and ARD mutants in transfected HEK293T cells was evaluated by immunofluorescence assay (IFA) using anti-HA antibody (Fig 8A). The average nucleus-to-cytoplasm (N/C) ratio was measured for each HBc mutant and calculated for each analyzed cell separately (Fig 8B). We found that predominant cytosolic localization of HBc required mutations in both putative NLS (ARDI and ARDIII). ARDI and ARDIII appeared to be equally strong nuclear import signals that acted co-dependently. This data is in agreement with already published study [13]. As for HBc nuclear export, it appeared that the mutation of a single ARDII (putative NES/CRS) was sufficient to induce strong nuclear localization of HBc, which could be further intensified by the mutation of ARDIV (putative NES/CRS). However, in the presence of *wt* ARDII, mutation of ARDIV had only minimal effect on accumulation of HBc in nucleus. Thus, ARDII seems to act as dominant nuclear export/cytoplasm retention signal, while ARDIV performs supportive role functionally dependent on the presence of primary NES/CRS—ARDII. According to Li et al. [13] only the double mutant ARDII_IV displayed a strong phenotype of nuclear localization. The minor difference between our and previously published data concerning the relative strength of NES/CRS could be due to the extent of the mutated region (three vs two R-to-A mutations), the choice of cells (HEK293T vs Huh7) or the system used for analysis (replicon vs. non-replicon).

### Effect of methylation on HBc subcellular localization

**Fig 9:**
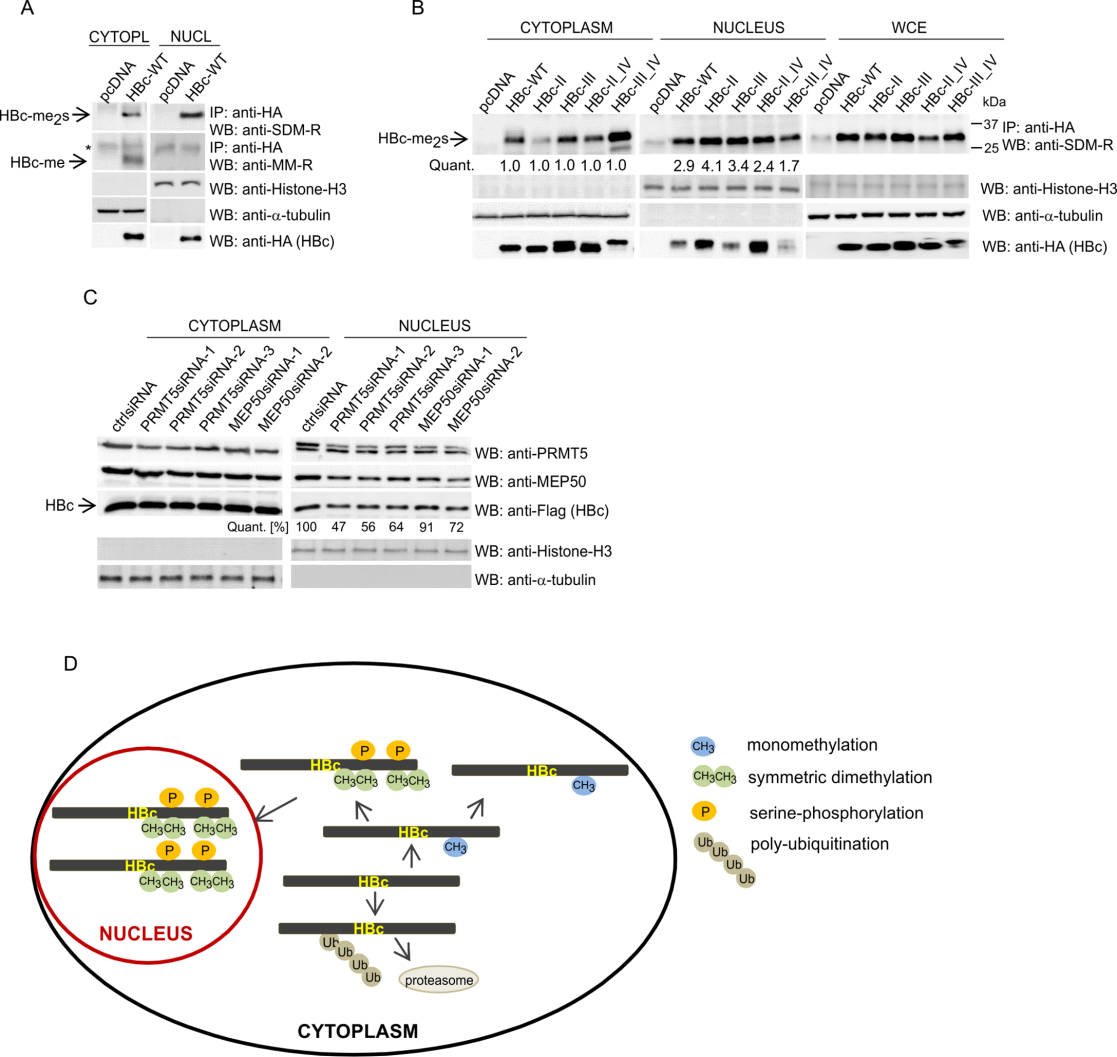
Nuclear versus cytoplasmic accumulation of monomethylated and symmetrically dimethylated HBc *wt* and ARD mutants. (A) HepG2_hNTCP cells were transfected with HA-tagged *wt*HBc or an empty vector, pcDNA, as indicated. Forty-eight hours after transfection, the cells were fractionated into nuclear and cytoplasmic extracts. The cell lysates (400 μg) were analyzed by immunoprecipitation (IP) with anti-HA-specific antibodies followed by Western blotting with SDM-R and MM-R antibodies. The relative levels of transfected HBc-HA in 40 μg of cell lysates were estimated by Western blots (bottom panel). *, non-specific WB signal. (B) HepG2_hNTCP cells were transfected with HA-tagged *wt*HBc and selected ARD mutants, as indicated. Forty-eight hours after transfection, the cells were fractionated into cytoplasmic (left), nuclear (middle) or whole-cell extracts (WCE; right). The cell lysates (400 μg) were analyzed by immunoprecipitation (IP) with anti-HA-specific antibodies followed by Western blotting with SDM-R antibodies. The relative levels of transfected HBc-HA in 40 μg of cell lysates were estimated by Western blots (bottom panel). *, non-specific WB signal. The intensity of methylation signal was quantified using the ImageQuant TL Array software and normalized to the level of HBc expression. The levels representing nuclear methylation of *wt* and individual ARD mutants were plotted against the corresponding levels of cytoplasmic methylation. (C) Down-regulation of PRMT5 and MEP50 leads to reduced levels of HBc protein in nuclei of transfected cells. HepG2_hNTCP_HBc-Flag stable cell line was transfected with PRMT5-, MEP50-specific siRNAs or control siRNA. Forty-eight hours after transfection, the cells were fractionated into nuclear and cytoplasmic extracts and analyzed by Western blot with anti-PRMT5, MEP50 and Flag antibodies. The intensity of HBc signal was quantified using the ImageQuant TL Array software and normalized to the level of Histone 3 expression. (D) A graphical representation of localization tendencies of different monomethylated, symmetrically dimethylated, phosphorylated and poly-ubiquitinated forms of HBc protein.

To determine the effect of methylation on the subcellular localization of HBc, we performed a cell fractionation assay in HepG2_hNTCP cells. The cells were transfected with *wt*HBc or an empty vector and forty-eight hours after transfection, the nuclear and cytoplasmic extracts were prepared. The cell fractionation assay indicated that symmetrically dimethylated HBc protein displayed an increased nucleus-to-cytoplasm (N/C) ratio (Fig 9A). Conversely, monomethylated HBc was exclusively localized in the cytoplasm. To determine the effect of NES or NLS mutations on intracellular distribution of symmetrically dimethylated HBc protein, we transfected HepG2_hNTCP cells with *wt*HBc or selected ARD mutants (II, III, II_IV, and III_IV) and the nuclear and cytoplasmic extracts were prepared. In agreement with IFA results (in Fig 8), the HBc mutants ARDII and ARDII_IV exhibited a significantly higher level of nuclear accumulation than *wt*HBc (Fig 9B, bottom panel). To analyze the subcellular distribution of symmetrically dimethylated HBc, the nuclear and cytoplasmic extracts were immunoprecipitated with anti-HA antibodies and analyzed by Western blot with anti-SDM-R (Fig 9B). While for *wt*HBc the N/C ratio of dimethylated HBc was approximately 2.9, for ARD mutants varied between 1.7 (for ARD mutant III_IV) to 4.1 (for ARD mutant II). This could also be partially influenced by mutations, which were introduced into putative NLS and NES regions.

We also examined the effect of PRMT5 and MEP50 silencing on HBc localization. To this end, a HepG2_hNTCP_HBc-Flag stable cell line was transfected with control siRNA, or PRMT5- and MEP50-specific siRNAs and after two days fractionized to nuclear and cytoplasmic extracts, and analyzed by Western blot with anti-Flag antibody. As shown in Fig 9C, down-modulation of PRMT5 and MEP50 reduced the levels of HBc protein in the nucleus indicating that inhibition of symmetric dimethylation may prevent nuclear import of HBc protein. In summary, our results suggested that arginine methylation could regulate the nucleus-to-cytoplasm shuttling of HBc protein and implicated PRMT5 in its control. Thus the symmetric dimethylation which is likely linked to serine phosphorylation could signal the nuclear import of HBc. Conversely, monomethylation would support accumulation of HBc in the cytoplasm (Fig 9D).

## Discussion

In this study we investigated the role of the type II arginine methyltransferase, PRMT5, in HBV transcription, replication, and methylation of HBc protein. HBc protein displays multiple pleiotropic functions, including the import of genetic material into cell nucleus, formation of HBV chromatin structure, protection of retrotranscribed genetic material in cytoplasm and export of virus particle during maturation process.

### HBc monomethylation versus symmetric dimethylation

Here, we have described arginine monomethylation and symmetric dimethylation as novel post-translational modifications of HBc protein. Mutational analysis of HBc ARDI to IV domains indicated that the presence of intact ARDI and ARDII is critical for efficient HBc methylation. Mass spectrometry and immunoprecipitation analyses identified two arginine residues, R150 and R156, which were modified by methylation. Although symmetrical dimethylation involved both arginine residues, the monomethylation predominantly occurred on R156. While R150 is located within the ARDI domain, arginine R156 is situated outside the ARD domains, in the region between ARDI and ARDII. Therefore, the lack of HBc methylation in ARDII mutants could be due to a conformational change near the methylation site or the disruption of HBc interaction domain with specific cellular methyltransferases, e.g. PRMT5.

One of the functional consequences of arginine methylation for many cellular proteins, including heterogeneous nuclear ribonucleoproteins (hnRNPs), is their relocalization within the cell. In mammalian cells, arginine methylation facilitates nuclear import or slowing of nuclear export [45]. Therefore, it is possible that symmetric dimethylation of HBc protein may represent a signal for nuclear import. Indeed, the majority of symmetrically dimethylated HBc accumulated in nucleus (Fig 9A and 9B).

The results presented here identified PRMT5 and possibly PRMT7 as cellular factors, which are involved in the methylation of HBc protein. Depletion of PRMT5 resulted in reduced levels of symmetrically dimethylated HBc. Conversely, overexpression of both PRMT5 and PRMT7 led to increased symmetric dimethylation of HBc. Therefore, it is possible, that PRMT5 together with PRMT7 may co-operate in the methylation of HBc. Indeed, PRMT7 was previously shown to interact with PRMT5 *in vivo* and both enzymes were implicated in the methylation of histone H3 Arg-2 and the Sm ribonucleoproteins [46, 47].

Based on presented data we proposed the model illustrating the interplay between monomethylation and symmetric dimethylation of HBc protein and its subcellular distribution (Fig 9D). According to this model, symmetric dimethylation is linked to increased serine phosphorylation and accumulation of HBc protein in nucleus. Contrary to this, monomethylated HBc retains in the cytoplasm.

### Potential effect of arginine methylation on HBc function

During the late phase of the viral life cycle, after HBV genome maturation, HBc facilitates cytoplasmic retention of nucleocapsids, their envelopment and egress of mature HBV particles from the cell. Concomitantly, a minor proportion of nucleocapsids is shuttled back to the nucleus to enable amplification of HBV cccDNA. Our results suggest that intracellular level of PRMT5 can control the nucleus-to-cytoplasm ratio. The C-terminal part of HBc protein has been shown to be “buried” in the capsid interior of icosahedral particles [48]. This may result in its inaccessibility for cellular factors involved in nucleus-to-cytoplasm shuttling and other HBc functions. Previous reports proposed the “charge balance hypothesis” of HBc capsid stability and assembly [49, 50]. According to this hypothesis, alterations of capsid conformation can be influenced by charge imbalance in the capsid inner surface. In a recent study, Su et al. [51] showed that serine phosphorylation at HBc ARD involving three major (S155, S162, S170 of ayw HBc) and four minor (S168, S176, S178 and S181 of ayw HBc) phosphorylation sites could considerably contribute to charge balance in the capsid interior. Thus HBV maintains electrostatic homeostasis by modulating the negative charges from phosphoserines and encapsidated nucleic acids [51]. Although methylation on arginine residues should not alter their charge, the extra bulk of the methyl groups could either inhibit protein binding or provide a new epitope for interaction. Thus, arginine methylation may control the binding of HBc to cellular factors, e.g. importins α and β, or NXF1-p15 [11, 13, 14, 43], that are involved in shuttling of HBc between nucleus and cytoplasm. The evidence, that HBc is subjected to multiple types of modifications, e.g. serine phosphorylation, ubiquitination, or arginine methylation, could suggest a potential cooperation among these different types of post-translational HBc modifications. Specific combinations of covalent modifications may disrupt or promote interactions of HBc with other cellular proteins and affect viral transport, encapsidation, or release.

### HBc and ubiquitin

As suggested by the results of mass spectrometry analysis and immunoprecipitation, HBc protein can also be modified by ubiquitination. Although modification of HBc by ubiquitination was implicated in several reports [52–54], the attempts to detect ubiquitinated HBc in cells were unsuccessful. It is of note that the HBc structural protein contains two potential ubiquitin acceptor lysine residues (K7 and K96) which are conserved across different HBV strains. K96 was suggested to serve as an ubiquitin conjugation site that aids viral replication and virion release [54, 55]. Although Garcia et al. [56] in a subsequent study questioned the importance of lysine K96 ubiquitination in viral replication, a mutation of K96 to arginine (K96R) altered the nuclear-cytoplasmic distribution of HBc, leading to its accumulation in the nucleolus. Importance in control of proteasome-mediated degradation and polyubiquitination of HBc protein was also suggested in recent studies. Qian et al. [52, 53] identified a novel E3 ubiquitin ligase, Np95/ICBP90-like RING finger protein (NIRF), that can bind to HBc and promote its degradation. Similar regulatory mechanism involving both mono- and polyubiquitination was described for influenza virus-encoded nucleoprotein, NP [57, 58]. While monoubiquitination of NP is crucial for viral RNA replication, polyubiquitination targets NP for degradation.

In the present study, we showed that lysine K7 can be covalently modified by ubiquitin. K-to-R mutation at position 7 resulted in decreased mono- and poly-ubiquitibnation levels of HBc. Nevertheless, as indicated by mass spectrometry data, other so-called non-canonical sites involving serine and threonine residues [59] could be potentially modified by ubiquitin. Detailed evaluation of non-canonical ubiquitination and understanding the precise role of HBc ubiquitination in virus replication would require further studies.

### Control of viral growth and replication by PRMTs

PRMT5 is a major symmetric arginine methyltransferase in mammals with one of the largest collection of substrates among the family of PRMTs. PRMT5-MEP50, along with PRMT7, play an important role in the splicing of mRNA through methylating spliceosomal proteins, Sm D1, D3 and B/B’ [46]. PRMT5 symmetrically dimethylates histones (e.g. H2AR3, H4R3, H3R2 and H3R8), which are linked to differential regulation of transcription [60]. In addition, the PRMT5 gene encodes several splicing variants (v1 to v6) which may exhibit different functions and subcellular distribution [61, 62]. Due to its pleiotropic nature, PRMT5 may affect HBV replication at almost all stages of viral life cycle, from epigenetic control of cccDNA, HBV RNA processing and nuclear export to pgRNA encapsidation, viral DNA synthesis and viral assembly. Some of these aspects of HBV regulation by PRMT5 were addressed in a recent study [35]. Zhang et al. [35] reported that PRMT5 restricts HBV replication by two mechanisms. Firstly, PRMT5 catalyzes the formation of H4R3me2s repressive mark on cccDNA minichromosome through interaction with HBc and chromatin remodeler Brg1. Secondly, PRMT5 inhibits HBV core particle production in a methyltransferase-independent manner by preventing interaction of pgRNA with viral polymerase.

The presented study further strengthened the restrictive role of PRMT5 in regulation of HBV replication and suggested a novel mechanism of PRMT5-mediated control of HBV core protein transport. According to our data, PRMT5 plays important role in symmetric dimethylation of HBc and thus regulates its intracellular trafficking. Since Zhang et al. implicated HBc in targeting PRMT5 to cccDNA, it is possible that PRMT5 directs HBc protein to nucleus in order to facilitate its interaction with cccDNA minichromosome.

A growing number of reports implicate cellular PRMTs and arginine methylation in control of virus growth and replication in both a positive and negative manner. PRMT1 has been shown to interact with HBx protein and inhibit HBV transcription [34]. PRMT6 can inhibit HIV-1 transcription through the methylation of Tat, Rev, and nucleocapsid proteins [63, 64]. PRMT6, but not mutant methyltransferase, significantly decreased Rev-mediated viral RNA export from the nucleus to the cytoplasm [64]. On the other hand, PRMT5 was shown to trigger the symmetric dimethylation of EBNA2 RG domain of EBV and coordinate EBNA2-mediated transcription [65]. PRMT1 plays a key role as a cellular activator of herpes simplex virus 1 replication through ICP27 RGG-box methylation [66]. Interestingly, E6 proteins of both low-risk and high-risk human papillomavirus (HPV) interact with three coactivator HMTs, CARM1, PRMT1 and SET7, and downregulate their enzymatic activities [67]. In summary, these studies together with our presented data underline the importance of PRMTs and arginine methylation in regulation of viral replication and may provide novel targets for future therapeutic interventions.

## Materials and methods

### Cell lines and culture conditions

HEK293T (human embryonic kidney) cells were obtained from ATCC and grown in Dulbecco's modified Eagle's medium supplemented with 10% fetal bovine serum (FBS). HepG2_hNTCP (a human liver cancer cell line, HepG2, stably transfected with the human HBV entry receptor—sodium taurocholate cotransporting polypeptide [hNTCP] was obtained from Dr. Stephan Urban [Heidelberg University Hospital, Heidelberg, Germany]) and HepG2.2.15 (a HepG2 cell line that harbors two head-to-tail dimers of the HBV genome [serotype ayw, genotype D; GenBank accession: U95551.1] was obtained from Dr. David Durantel [Cancer Research Center of Lyon, Lyon, France]), were grown in Dulbecco's modified Eagle's medium supplemented with 10% FBS and puromycin (0.05 mg/ml) or G418 (0.4 mg/ml), respectively. HepG2_hNTCP cells constitutively expressing HBc-Flag (HepG2_hNTCP_HBc-Flag) were generated by transfection of HepG2_hNTCP cells with Flag-tagged HBc-expressing plasmid, and the transfected cells were selected by growth in G418 (0.8 mg/ml).

### Plasmids

Expression plasmids for PRMT1, PRMT3, PRMT5v1 (splice variant 1), PRMT5v2 (splice variant 2), PRMT7, PRMT9 (Q6P2P2 in the UniProt Database, previously also referred to as PRMT10) and MEP50/WDR77 tagged with myc and Flag were purchased from OriGene Technologies. The full-length HBc (1–185 aa, Genotype A, subtype adw2) expression plasmids, HBc-HA or HBc-Flag, HBc-V5/AP and GST-HBc, were generated by PCR amplification of HBc ORF (as a template we used plasmid pHY92CMV obtained from Dr. Huiling Yang [Gilead Sciences, Inc., Foster City, USA]) followed by subcloning into pcDNA3.1 (ThermoFisher), the Gateway destination vector pcDNA-3.2/capTEV-CT/V5-DEST (ThermoFisher) and pGEXT4 (GE Healthcare Life Sciences), respectively. The C-terminal truncations of HBc (aa 1–149), HBc-ΔC-HA and GST-HBc-ΔC, were generated by PCR amplification of HBc ORF corresponding to aa 1–149 and subsequently cloned into pcDNA3.1 or pGEXT4 vectors, respectively. The R-to-A and K-to-R mutants of HBc were generated by PCR, using QuickChange XL Site-Directed Mutagenesis (Agilent Technologies). In all HA- or Flag-tagged HBc constructs, the tags were fused to the C-terminus of HBc protein. The fidelity of all constructs was verified by sequencing (GATC Biotech AG).

### siRNAs

The PRMT-specific siRNAs utilized were as follows: PRMT1siRNA (s6917, ThermoFisher), PRMT3siRNA (s19869, ThermoFisher), PRMT5siRNA-1 (targets exon 8, s20375, ThermoFisher), PRMT5siRNA-2 (targets exon 7, SASI_Hs01_00182741, Sigma Aldrich) and PRMT5siRNA-3 (targets exon 11, SASI_Hs02_00340905, Sigma Aldrich). All three PRMT5-specific siRNAs were designed to target the majority of PRMT5 splicing variants. The MEP50-specific siRNAs, MEP50siRNA-1 (SASI_Hs01_00203545), and MEP50siRNA-2 (SASI_Hs01_00203544), were purchased from Sigma Aldrich and ctrlsiRNA (non-targeting siRNA, 4390843) was obtained from ThermoFisher. The specificity and inhibitory potential of PRMT-siRNAs was estimated by RT-qPCR using the following primer pairs: PRMT1-F 5'-TGCGGTGAAGATCGTCAAAGCC-3', PRMT1-R 5'-GGACTCGTAGAAGAGGCAGTAG-3'; PRMT3-F 5'-CACTGTCTGCTGAAGCCGCATT-3', PRMT3-R 5'-GTAGATGACGAGCAGGTTCTGAC-3'; PRMT5-F 5'-CTAGACCGAGTACCAGAAGAGG-3', PRMT5-R 5'-CAGCATACAGCTTTATCCGCCG-3'; and MEP50-F 5'-CTCAGGTCACTTGTGTTGCTGC-3', MEP50-R 5'-ATCTGTGATGCTGGCTTGGGAC-3'. RNA was reverse-transcribed using AccuScript Hi-Fi Reverse Transcriptase (Agilent Technologies) and the corresponding antisense (R) primer. qPCR was performed with TaqPlatinum Polymerase (ThermoFisher Scientific) and SYBR Green dye (Sigma). The 25 μl qPCR mixture consisted of 5 μl cDNA, primers (0.2 μM each), 0.2 mM dNTPs, 5 mM MgCl_2_, 1.25 U Platinum *Taq* DNA polymerase, 1 × PCR buffer and SYBR Green dye diluted 1: 2,500. The following thermal cycling conditions for qPCR run were used: denaturation (95° C for 10 min), denaturation and annealing/elongation steps repeated 40 times with fluorescence measurement at the end of the elongation step (95°C for 15 s, 60° C for 1 min), melting curve program (60°–95°C with a heating rate of 0.1°C per second and a continuous fluorescence measurement). The levels of PRMTs’ mRNAs were normalized to β-actin. For amplification of β-actin cDNA, the following primers were used: β-actin-F 5'-CTCTTCCAGCCTTCCTTCCT-3', β-actin-R 5'-AGCACTGTGTTGGCGTACAG-3'.

### Antibodies

The primary antibodies and reagents used for Western blot and immunoprecipitations include rabbit polyclonal anti-HBc antibodies (Dako), mouse monoclonal anti-HBc (clone Hyb3120, CosmoBio), anti-β-actin (Abcam), rabbit monoclonal anti-PRMT5 (Abcam), anti-MEP50/WDR77 (Abcam), anti-histone H3 (Abcam), anti-alpha-tubulin (Abcam), anti-phosphoserine (Abcam), anti-symmetric dimethyl arginine (SDM-R) (Cell Signaling), anti-monomethyl arginine (MM-R) (Cell Signaling), anti-HA (Santa Cruz), anti-Ubiquitin (Cell Signaling), anti-SmB/B’ (Sigma), mouse anti-Flag (Sigma-Aldrich), anti-HA magnetic beads (ThermoFisher), and anti-Flag magnetic beads (Sigma). Secondary antibodies include goat polyclonal anti-mouse horseradish peroxidase (HRP) (Sigma-Aldrich) and goat polyclonal anti-rabbit-HRP (Sigma-Aldrich). Antibodies for immunofluorescence analysis (IFA) include mouse monoclonal anti-HA antibody conjugated with FITC (Sigma-Aldrich).

### HBV virions

HBV virions were produced from HepG2.2.15 cells grown in DMEM medium containing 10% FBS and 2% dimethyl sulfoxide (DMSO). HBV particles were precipitated from clarified cell supernatants by overnight incubation in 6% polyethylene glycol 8000 (PEG 8000) and were then concentrated by centrifugation at 4°C for 90 min at 14,000 x g. The pelleted HBV was suspended in complete DMEM medium supplemented with 10% FBS. HBV titers were determined by quantitative PCR (qPCR) using primers specific for HBV DNA: HBV-F 5'-AGAGGACTCTTGGACTCTCTGC-3'; HBV-R 5'-CTCCCAGTCTTTAAACAAACAGTC-3', and the probe pHBV 5'-[FAM]TCAACGACCGACCTT[BHQ1]-3'. qPCR was performed with gb Elite PCR Master Mix (Generi Biotech) and TaqMan probe. The 20 μl qPCR mixture consisted of 5 μl DNA, primers (0.3 μM each), probe (0.1 μM) and 1 × gb Elite Master Mix. Thermal cycling conditions for qPCR run were: denaturation (95°C for 10 min), denaturation and annealing/elongation steps repeated 40 times with fluorescence measurement at the end of the elongation step (95°C for 15 s, 60°C for 1 min).

### HBV infection and analysis of HBV replication

The HBV infection of transfected HepG2_hNTCP cells (1000 viral genome equivalents per cell [VGE/cell]) was done overnight in the presence of 4% PEG8000 and 2.5% DMSO. Four days post-infection, the cells were harvested for RNA isolation using RNeasy Plus Mini Kit (Qiagen). Contaminating genomic DNA was removed by treatment with DNase I Amplification grade (ThermoFisher Scientific). RNA integrity was evaluated by agarose gel electrophoresis. One microgram of RNA was reverse-transcribed using SuperScript VILO cDNA synthesis kit (ThermoFisher Scientific). The HBV pgRNA was amplified by qPCR using the following primer sets: pgRNA-F 5'-GGTCCCCTAGAAGAAGAACTCCCT-3'; pgRNA-R 5'-CATTGAGGTTCCCGAGATTGAGAT-3'; pgRNA probe 5'-[FAM]TCTCAATCGCCGCGTCGCAGA[BHQ1]-3′. The pgRNA primers can also amplify HBV preC RNA. The levels of pgRNA+preC were normalized to β-actin and the pgRNA+preC to cccDNA ratio was calculated. Four days post-infection, the cells were harvested for DNA isolation using QIAamp DNA Blood Mini Kit (Qiagen). The levels of total HBV DNA in cells were analyzed by qPCR using an Eppendorf realplex Master cycler and gb Elite PCR Master Mix (Generi Biotech). The primer/probe set for HBV DNA (HBV-F, HBV-R and pHBV) quantification is listed above. The cccDNA quantification (for detailed protocol see dx.doi.org/10.17504/protocols.io.je8cjhw) was done as described previously [68]. Briefly, the isolated DNA was subjected to T5 exonuclease (NEB) treatment at 37°C for 30 min, followed by heat inactivation at 99°C for 5 min. Following treatment, the cccDNA was diluted fourfold and analyzed by qPCR using the following primers and probe: cccDNA-F 5′-CCGTGTGCACTTCGCTTCA-3′, cccDNA-R 5′-GCACAGCTTGGAGGCTTGA-3′ and cccDNA probe 5′-[FAM]CATGGAGACCACCGTGAACGCCC[BHQ1]-3′. The validation of cccDNA amplification was performed with serial dilutions of HBV rcDNA samples. The levels of HBV DNA and cccDNA were normalized to albumin (Alb-F 5'-GCTGTCATCTCTTGTGGGCTGT-3'; Alb-R 5'-AAACTCATGGGAGCTGCTGGTT-3'; and Alb-probe 5'-[FAM]GGAGAGATTTGTGTGGGCATGACAGG[BHQ1]-3').

### HBsAg and HBeAg detection by ELISA

The titers of HBsAg and HBeAg were quantified by ELISA. HepG2_hNTCP cell culture supernatants at day 4 post-infection from each experimental group were collected and centrifuged at 120 × g for 10 min to remove cellular debris, transferred to clean tubes and stored at -80°C until antigen measurement. The titers of HBsAg and HBeAg were measured using a commercial ELISA kit (Bioneovan, Beijing, China) according to the manufacturer’s instructions.

### Transfections

All transfections of expression plasmids were carried out using Lipofectamine 2000 (HEK293T cells) or Lipofectamine 3000 (HepG2_hNTCP cells) transfection reagents (Life Technologies). The HepG2_hNTCP cells were transfected (in 24-well plates, 6-well plates or 10 cm plates) with PRMT1, 3, 5, 7, 9 MEP50/WDR77, HBc expression plasmids or an empty vector—pcDNA.

### Immunofluorescence analysis (IFA)

Transiently transfected HEK293T cells were grown on 22-mm glass coverslips for 24 h, briefly washed with phosphate-buffered saline (PBS) and then fixed with 4% paraformaldehyde in PBS for 30 min at room temperature. Fixed cells were washed with PBS and permeabilized with 0.2% Triton X-100 in PBS for 30 min. Permeabilized cells were immunostained in PBS, 0.2% Triton X-100, 10% FBS for 1 h using an appropriate primary antibody. The cells were washed three times for 10 min with 0.2% Triton X-100 in PBS. After final washes, the immunostained coverslips were mounted on slides in ProLong Diamond Antifade Mountant with DAPI (Thermo Fisher Scientific). Images were acquired with a three-dimensional microscopy system Zeiss LSM 780 (Carl Zeiss) using a 63× oil objective with a numerical aperture of 1.4. Images were collected with a pinhole at diameter 0.7 μm (1 Airy unit), averaged four times and processed with ZEN 2011 software (Carl Zeiss). Single cells quantification experiments were performed on individual channel images exported from ZEN 2011 as.tiff files using multidimensional image data processing software ImageJ (NIH). Subcellular localization was examined individually on at least 10 HBc-positive cells representative for each sample. For HBc ARD mutants (ARDIII, III_IV, I_II_III_IV) which displayed heterogeneous phenotypes, more than 30 single cells were analyzed.

### GST pull-down

*In vitro*-translated PRMT1, 3, 5 and MEP50 were synthesized using a TNT T7 quick-coupled transcription/translation system (Promega) according to the manufacturer's instructions. GST-HBc, GST-HBc-ΔC fusion proteins or GST alone (0.5 μg) bound to glutathione-Sepharose beads (GE Healthcare Life Sciences) were incubated with 3 to 10 μl of the reaction mixture consisting of *in vitro*-translated proteins in 500 μl of binding buffer (10 mM Tris [pH 7.6], 100 mM NaCl, 0.1 mM EDTA [pH 8.0], 1 mM dithiothreitol, 5 mM MgCl_2_, 0.5% Igepal CA-630, 8% glycerol, 0.2 mM protease inhibitor mixture [Sigma-Aldrich]) at 4°C for 3 h. After five 10-min washes with binding buffer supplemented with 1% Igepal CA-630, the proteins that were bound to the beads were analyzed by Western blotting with anti-Flag-specific antibodies.

### Immunoprecipitation analysis and Western blot

(For detailed protocols see dx.doi.org/10.17504/protocols.io.je8cjhw). For HBc co-immunoprecipitation experiments, the cells were transfected with HBc-expression plasmid, HBc-V5/AP, together with plasmids expressing human PRMTs and MEP50 tagged with Flag. The expression plasmid HBc-V5/AP contains a V5 tag and also enables target protein biotinylation by the eukaryotic cellular machinery during expression. Forty-eight hours after transfection, the cells were lysed in co-immunoprecipitation buffer (20 mM HEPES [pH 7.9], 50 mM NaCl, 5 mM EDTA, 1% (v/v) Igepal CA-630, 10% (v/v) glycerol, 1 mM dithiothreitol, 1 mM phenylmethylsulfonyl fluoride (PMSF), and 0.2 mM protease inhibitor cocktail [all Sigma-Aldrich]) and protein extracts (400 μg) were incubated with anti-Flag (Sigma-Aldrich) magnetic beads at 4°C overnight. Immune complexes were extensively washed with co-immunoprecipitation buffer and co-precipitated HBc-V5/AP was detected by Western blotting with NeutrAvidin-HRP (Life Technologies). For detection of HBc arginine methylation and serine-phosphorylation, the cells were harvested in lysis buffer (150 mM NaCl, 5 mM EDTA, 1% Triton X-100, 10 mM Tris–HCl, pH 7.5, 50 mM NaF, 1 mM PMSF, 100 μM NaVO_3_ [all three Sigma-Aldrich] and 0.2 mM protease inhibitor cocktail). The protein extracts were incubated with anti-HA magnetic beads (ThermoFisher) at 4°C for 4 h. The immune complexes were extensively washed with the lysis buffer and analyzed by Western blotting with anti-SDM-R, anti-MM-R, or anti-phosphoserine antibodies. For detection of HBc ubiquitination (Figs 6A, 7A and 7B), the cells were lysed in 2% SDS, 150 mM NaCl, 10 mM Tris-HCl, pH 8.0) with 2mM sodium orthovanadate, 50 mM sodium fluoride, 10 mM N-ethylmaleimide (NEM), and protease inhibitors, followed by incubation at 100 °C for 10 min and sonication. The lysates were then diluted with 9 volumes of dilution buffer (10 mM Tris-HCl, pH 8.0, 150 mM NaCl, 2 mM EDTA, 1% Triton) and subjected to immunoprecipitation with anti-Flag or anti-HA magnetic beads (ThermoFisher). After 4-hour incubation at 4°C, the precipitates were washed with the washing buffer (10 mM Tris-HCl, pH 8.0, 1 M NaCl, 1 mM EDTA, 1% NP-40) and analyzed by Western blotting with anti-Ub antibodies. Nuclear and cytoplasmic protein fractions were isolated using NE-PER nuclear and cytoplasmic extraction kit (ThermoFisher) according to manufacturer’s recommendations. The intensity of Western blot signals was quantified using the ImageQuant TL Array software (GE Healthcare Life Sciences).

### PRMT5 splice variants

To analyze the expression of alternatively spliced variants of PRMT5 in HepG2_hNTCP, we used semi-quantitative RT-PCR approach. HepG2_hNTCP cells were harvested for RNA isolation using RNeasy Plus Mini Kit (Qiagen). Contaminating genomic DNA was removed by treatment with DNase I Amplification grade (ThermoFisher). RNA integrity was evaluated by agarose gel electrophoresis. One microgram of RNA was reverse-transcribed using SuperScript III First-Strand Synthesis System for RT-PCR (ThermoFisher Scientific) followed by PCR with Phusion High Fidelity DNA Polymerase (New England BioLabs) and primers specific for 5’UTR and 3’UTR of PRMT5 mRNA: 5’UTR-F 5'-GGCGTGGACAGCGCGAGGAG-3'; 3’UTR-R 5'-CAAGGCTCTGGACACTTGGC-3'. The 50 μl PCR mixture consisted of 5 μl cDNA, primers (0.4 μM each), 0.2 mM dNTPs, 0.5 mM MgCl_2_, 1 U Phusion High-Fidelity DNA Polymerase and 1 × Phusion HF buffer. PCR conditions consisted of initial denaturation (98°C / 1 min) and 20 cycles with denaturation (98°C / 30 s), annealing (55°C / 30 s) and elongation (72°C / 3 min). The PCR products were cloned using TOPO TA Cloning Kit (ThermoFisher Scientific) following the manufacturer’s instructions and 30 clones were sequenced and analyzed.

### Mass spectrometry analysis (LC-MS/MS)

(For details see dx.doi.org/10.17504/protocols.io.je8cjhw). The SDS-PAGE protein gel stained by coomassie blue was divided into 14 slices. Gel slices were distained with 25 mM ammonium bicarbonate in 50% acetonitrile at 30°C for 30 min and dried with 200 μl acetonitrile for 5 min at 30°C. Dry gel pieces were treated with dithiothreitol (65°C, 30 min) and iodoacetamide (RT for 30 min in dark) to reduce and alkylate cysteines. Proteins in gel pieces were digested with 0.1 μg of trypsin solution in 50 mM ammonium bicarbonate at 37°C for 10 h. Peptides were extracted with 50 μl of 2% TFA and 50 μl of 60% acetonitrile. The peptides were dried in the SpeedVac and dissolved in 15 μl of 0.1% formic acid.

Immunoprecipitated proteins on magnetic beads were washed 3x with 1 ml of 50 mM sodium hydrocarbonate. Proteins in the sample were reduced by dithiothreitol (65°C, 30 min) and alkylated by iodoacetamide (RT for 30 min in dark). Solvent was removed, 100 ul of 50 mM sodium hydrocarbonate including 0.1 μg of chymotrypsin were added and proteins were digested at 37°C for 10 h. Resulting peptides were removed from beads by magnetic separation, dried in the SpeedVac (Labconco) and dissolved in 15 μl of 0.1% formic acid.

All samples were analyzed on UltiMate 3000 RSLCnano system (Dionex) coupled to a TripleTOF 5600 mass spectrometer with a NanoSpray III source (Sciex). The instrument was operated with Analyst TF 1.7 (Sciex). The peptides were trapped and desalted with 2% acetonitrile in 0.1% formic acid at flow rate of 5 μL/min on Acclaim PepMap100 column (5 μm, 2 cm×100 μm ID, Thermo Scientific). Eluted peptides were separated using Acclaim PepMap100 analytical column (3 μm, 25 cm×75 μm ID, Thermo Scientific). The 70 min elution gradient at constant flow of 300 nl/min was set to 5% of phase B (0.1% formic acid in 99.9% acetonitrile, phase A 0.1% formic acid) for first 5 min, then with gradient elution by increasing content of acetonitrile. TOF MS mass range was set to 350–1500 m/z, in MS/MS mode the instrument acquired fragmentation spectra with m/z ranging from 100 to 2000.

Protein Pilot 4.5 (Sciex) search engine was used for protein identification from raw (*.wiff) spectra using database consisting of protein variants of HBV proteins and their ARD mutants, human proteins and common contaminants (Source–Uniprot). The search was set to choose iodoacetamide as alkylation substance, trypsin as digestion agent and TripleTOF 5600 as instrument. All samples were evaluated by Paragon algorithm in the regime „Thorough“. Set of biological modification as defined by the company with different probabilities of potential modifications was employed. The mass spectrometry proteomics data have been deposited to the ProteomeXchange Consortium via the PRIDE [69] partner repository with the dataset identifier PXD006828.

### Statistical analysis

Results in graphs are expressed as mean ± SD (standard deviation). To compare differences between experimental groups and control, we used analysis of variance test (ANOVA) followed by the Dunnett’s test for multiple comparisons. Data were analyzed with GraphPad Prism v.6.05 (GraphPad Software). The symbols ***, **, and * were used when P<0.001, P<0.01, and P<0.05, respectively.
